# Led-Seq: ligation-enhanced double-end sequence-based structure analysis of RNA

**DOI:** 10.1093/nar/gkad312

**Published:** 2023-04-28

**Authors:** Tim Kolberg, Sarah von Löhneysen, Iuliia Ozerova, Karolin Wellner, Roland K Hartmann, Peter F Stadler, Mario Mörl

**Affiliations:** Institute for Biochemistry, Leipzig University, Brüderstr. 34, 04103 Leipzig, Germany; Bioinformatics Group, Department of Computer Science and Interdisciplinary Center for Bioinformatics, Leipzig University, Härtelstr. 16–18, 04107 Leipzig, Germany; Bioinformatics Group, Department of Computer Science and Interdisciplinary Center for Bioinformatics, Leipzig University, Härtelstr. 16–18, 04107 Leipzig, Germany; Institute for Biochemistry, Leipzig University, Brüderstr. 34, 04103 Leipzig, Germany; Institute for Pharmaceutical Chemistry, Philipps University Marburg, Marbacher Weg 6, 35037 Marburg, Germany; Bioinformatics Group, Department of Computer Science and Interdisciplinary Center for Bioinformatics, Leipzig University, Härtelstr. 16–18, 04107 Leipzig, Germany; Max Planck Institute for Mathematics in the Sciences, Inselstraße 22, D-04103 Leipzig, Germany; Department of Theoretical Chemistry, University of Vienna, Währingerstraße 17, A-1090 Wien, Austria; Facultad de Ciencias, Universidad Nacional de Colombia, Bogotá, Colombia; Santa Fe Institute, 1399 Hyde Park Rd., Santa Fe, NM 87501, USA; Institute for Biochemistry, Leipzig University, Brüderstr. 34, 04103 Leipzig, Germany

## Abstract

Structural analysis of RNA is an important and versatile tool to investigate the function of this type of molecules in the cell as well as *in vitro*. Several robust and reliable procedures are available, relying on chemical modification inducing RT stops or nucleotide misincorporations during reverse transcription. Others are based on cleavage reactions and RT stop signals. However, these methods address only one side of the RT stop or misincorporation position. Here, we describe Led-Seq, a new approach based on lead-induced cleavage of unpaired RNA positions, where both resulting cleavage products are investigated. The RNA fragments carrying 2′, 3′-cyclic phosphate or 5′-OH ends are selectively ligated to oligonucleotide adapters by specific RNA ligases. In a deep sequencing analysis, the cleavage sites are identified as ligation positions, avoiding possible false positive signals based on premature RT stops. With a benchmark set of transcripts in *Escherichia coli*, we show that Led-Seq is an improved and reliable approach based on metal ion-induced phosphodiester hydrolysis to investigate RNA structures *in vivo*.

## INTRODUCTION

RNA is an extremely versatile molecule, despite its limited number of building blocks. It performs various tasks in many biological processes ranging from encoding genetic information as mRNAs to delivering amino acids to the ribosome as tRNAs, catalyzing chemical reactions, regulating gene expression and shielding viral RNA from degradation by host RNases, just to name examples (1–3). The functional diversity of RNA is based on helical arrangements comprising stacking interactions and base pairing that form both local structural motifs and long range interactions (4).

RNA structure formation is energetically dominated by canonical (Watson–Crick and GU-wobble) pairs forming helical stem regions separated by unpaired stretches of nucleotides. Such secondary structures also appear as intermediates during the folding process before additional interactions stabilize the three-dimensional structure (5). RNA secondary structure, i.e. the arrangement of canonical base pairs, can be computed based on an energy model that considers sequence-specific stacking of adjacent base pairs and entropy-driven, destabilizing contributions of loops (6). Efficient dynamic programming algorithms have been devised that compute minimum free energy (MFE) structures (7) and partition functions (8). Deviations from this simple nearest neighbor model, inaccuracies of energy parameters in particular large and multi-branched loops, tertiary interactions including pseudoknots, the limited understanding of the effects of salt concentrations and temperature limit the accuracy of the thermodynamic model. Additional factors such as interactions with proteins, metal ions and other ligands as well as the cellular localization of RNA molecules likewise have an impact on the conformational states (9,10). That makes computational prediction difficult and highlights the need for additional data to infer reliable RNA structures. Chemical probing methods provide information on the base pairing status of individual nucleotides. While it is well known that this information alone is insufficient to uniquely determine a secondary structure, it can be readily combined with thermodynamic prediction algorithms in the form of position-specific constraints or ‘bonus energies’ to guide the reconstruction of the biologically relevant structure (11–13). It is crucial, therefore, to develop and improve methods to determine RNA structure *in vivo* to deepen our understanding of how it is forming and how this structure can be predicted.

A rapid development has taken place in the last two decades regarding the determination of RNA secondary and tertiary structure (reviewed in (14–16)). Although biophysical methods such as X-ray crystallography, NMR and cryo-EM also have the potential to provide detailed insights into RNA structure, they are limited to *in vitro* applications. The possibility to structurally investigate the entirety of RNAs *in vivo* was pioneered with the development of SHAPE (17). This method and its derivatives evolved over the years utilizing different reagents, like NMIA, 1M7 or BzCN, to modify RNA depending on its structure (18). The coupling of SHAPE and other previously established structure probing methods with capillary electrophoresis and later high throughput sequencing allowed massively parallel investigation of RNA structures, enabling a global assessment of the ‘*in vivo* structurome’ (19–21). Most methods target nucleotides in single-stranded and conformationally flexible RNA regions that are more accessible to chemical modification than base-paired regions. Chemical modification gives rise to either reverse transcription (RT) stop (-seq approaches) or mutation signals (-MaP approaches) in cDNA. These signals can be read out as a reactivity score and be used to infer RNA structure (10). Structure-dependent cleavage is an attractive alternative to the covalent modification strategy. Compared to enzymatic cleavage, which is to date limited to *in vitro* applications (22,23), divalent metal ions promise to be an interesting alternative for *in vivo* investigations.

In particular Pb^2+^ is well established in RNA structure probing both *invitro* and *invivo* (24). Briefly, this method relies on structure dependent cleavage catalyzed by hydrated Pb^2+^ ions that abstract the proton from the ribose 2′-OH group. In an in-line configuration, the 2′-O^−^ group attacks the phosphorous in the sugar-phosphate backbone, resulting in cleavage of the RNA with fragments carrying 2′,3′-cyclic phosphate (2′,3′-cP) and 5′-OH ends. As flexible regions are more likely to adopt the in-line conformation, this reaction occurs predominantly in single-stranded regions. Enhanced cleavage can also occur at metal ion binding sites (25,26). Lead-seq (27) combines the established *in vivo* Pb^2+^ probing approach (28) with next generation sequencing (NGS) to allow its use in a high-throughput setting. It serves as a promising starting point to broaden the spectrum of methods to investigate the RNA structurome *in vivo*. The incorporation of a lead cleavage score as ‘bonus energy’ in the minimum free energy structure computation resulted in a distinct improvement of the predicted structure of several tRNAs. Like most NGS-coupled probing approaches, Lead-seq uses random fragmentation to generate a more homogeneously sized pool of RNAs. This introduces additional strand breaks, which may obscure the actual cleavage signal even though a negative control can reduce this effect to a certain degree. The 5′ phosphorylation and 3′ dephosphorylation of the RNAs with T4 polynucleotide kinase prior to adapter ligation impedes the distinction of the 5′ and 3′ phosphorylation status of the ligated RNAs. In this manner, all cellular RNAs are potentially included in the libraries, which negatively impacts the specificity of the method. Lead-seq thus is a promising concept with ample potential for improvement.

Here we propose a modified procedure that improves the specificity and validity of this method. Cleavage by divalent metal ions introduces strand breaks that generate fragments with distinct end groups, 2′,3′-cP and 5′-OH. For the specific capture of these fragments, we utilized the unique features of two RNA ligases to mark the cleavage positions via ligation of sequencing adapters. The 5′ fragments, carrying a 2′,3′-cP, are captured by an *Arabidopsis thaliana* tRNA ligase (*Ath* RNL) variant. The wild-type enzyme is able to ligate 2′,3′-cP and 5′-OH via its 2′,3′ cyclic phosphodiesterase and 5′ kinase activity (29,30). To ensure specific ligation of a pre-adenylated adapter to the substrate RNA, two mutations were introduced that inactivate the enzyme’s ability to phosphorylate and adenylate 5′-OH groups (31). This *Ath* RNL AA double mutant recently proved to keep its specificity for 2′,3′-cP carrying substrates in a ribozyme activity screen (32). The corresponding 3′ fragments, carrying 5′-OH ends, are captured in a similar manner utilizing *Escherichia coli* (*Eco*) RtcB. This ligase and its homologs also have the ability to directly ligate 5′-OH and 2′, 3′-cP but exhibit a unique mechanism without the necessity of phosphorylating the RNA 5′ end (33). It also proved to be applicable to library preparation in previous studies (34,35). The combination of two separate ligation approaches and analysis of cleavage positions from both sides allows mutual validation of the identified cleavage sites. Moreover, the use of reads from both the 5′ and 3′ side ensures that one of the libraries remains informative close to the transcript ends, where the reads from the other library are too short for unambiguous mapping.

## MATERIALS AND METHODS

### Protein purification

*Ath* RNL K152A D726A was expressed with 6xHisTag from a pET28a vector kindly gifted by Christina Weinberg in *E. coli* BL21 (DE3) codon PLUS RIPL cells. *Saccharomyces cerevisiae* Tpt1 was expressed from a pET-24b vector in *E. coli* BL21 (DE3) cells. Both enzymes were expressed and purified as described (32). T4 RNL 2 truncated KQ and TS2126 RNL expression plasmids were gifted by Jan Medenbach and the proteins were expressed and purified as described previously (36,37). *Eco* RtcB was expressed with 6xHisTag from a pET-53 vector (Addgene #51282) in *E. coli* BL21 (DE3) according to (38). Briefly, transformed cells were cultivated in TB medium with 100 μg/ml ampicillin at 37°C to an OD_600_ of 0.6. Cultures were chilled on ice for 30 min before induction with 0.1 mM IPTG and addition of ethanol to a final concentration of 2%. The induced cells were cultivated for 18 h at 16°C and harvested by centrifugation. Pellets were stored at −80°C until use. Cell pellets were resuspended in 25 ml ice-cold lysis buffer (50 mM Tris–HCl, pH 7.4, 250 mM NaCl, 10% (w/v) sucrose, 0.2 mg/ml lysozyme) and incubated for 1 h at 4°C. Then Triton-X100 was added to a final concentration of 0.1%. Cells were disrupted by sonication at 70% intensity, 7 × 10 s with 20 s breaks. The lysate was centrifuged, the supernatant sterile-filtered and used for purification. All further purification steps were carried out on an ÄKTA pure protein purification system starting with metal ion affinity chromatography on a HisTrapFF 1 ml column (Cytiva). To this end, the column was equilibrated with 5 column volumes (CV) of binding buffer (50 mM Tris–HCl, pH 7.4, 150 mM NaCl, 10% glycerol) containing 25 mM imidazole. After loading the sample, the column was washed with 10 CV binding buffer with 25 mM imidazole, followed by a second wash step with 10 CV wash buffer (50 mM Tris–HCl, pH 7.4, 2 M KCl). Step-wise elution was carried out using 5 CV binding buffer containing 100, 300 and 500 mM imidazole. Fractions containing *Eco* RtcB ligase were identified via SDS-PAGE and Coomassie staining, pooled and subsequently purified by size exclusion chromatography on a HiLoad 16/60 Superdex 75 pg column with SEC buffer (10 mM Tris–HCl, pH 8.0, 350 mM NaCl, 1 mM DTT). Fractions containing the desired protein were identified via SDS-PAGE, pooled and concentrated with a Vivaspin 6 column (MWCO 10 kDa) and stored at −80°C until use.

T4 polynucleotide kinase R38A was expressed with 6xHisTag from a pET-53 vector in *E. coli* BL21 (DE3) according to (39). Transformed cells were grown in TB medium containing 100 μg/ml ampicillin at 37°C to an OD_600_ of 0.6. The cultures were chilled on ice for 30 min before induction with 0.3 mM IPTG and cultivation at 16°C for 18 h. Cells were harvested via centrifugation and stored at −80°C until use. The cells were resuspended in 25 ml ice-cold lysis buffer (50 mM Tris–HCl, pH 7.5, 1.2 M NaCl, 15 mM imidazole, 10% (v/v) glycerol, 0.2 mM phenylmethylsulphonyl fluoride, 1 mg/ml lysozyme) and incubated 1 h at 4°C. Triton-X100 was added to a final concentration of 0.1%. Cells were disrupted by sonication at 70% intensity, 7 × 10 s with 20 s breaks. The lysate was centrifuged, the supernatant sterile-filtered and used for purification. Metal ion affinity chromatography was carried out on an ÄKTA pure protein purification system using a HisTrapFF 1 ml column (Cytiva). The binding buffer contained 50 mM Tris–HCl, pH 7.5, 200 mM NaCl and 10% glycerol. For step-wise elution, the 5 CV buffer additionally contained 125, 300 and 500 mM imidazole. The fractions with T4 PNK R38A were identified by SDS-PAGE, subsequently pooled and dialyzed twice against 1 l dialysis buffer (10 mM Tris–HCl, pH 7.4, 50 mM KCl, 1 mM DTT). Glycerol mix was added to reach storage conditions (10 mM Tris–HCl, pH 7.5, 50% (v/v) glycerol, 0.2 mM EDTA, 1 mM DTT, 50 mM KCl, 0.2 μM ATP). Aliquots of the protein solution were stored at –80°C. Plasmids encoding the enzymes used in this work are either available at Addgene (addgene.org) or from the authors upon request.

### Oligonucleotide preparation

Oligonucleotides were ordered from biomers (Ulm, Germany) and Microsynth (Balgach, Switzerland). 3′ Adapter, RT-Primer and circularization RT-Primer were 5′ labeled using [γ^32^P]-ATP and T4 PNK (NEB) and purified via denaturing PAGE and ethanol precipitation. Non-radioactively labeled adapters and RT-Primers were prepared in the same way with ATP and T4 PNK, using T4 PNK (3′ phosphatase minus) for the 5′ adapter. The 5′ phosphorylated and 3′ blocked 3′ adapter was pre-adenylated with TS2126 RNL. In one 20 μl reaction, 2.5 μM TS2126 RNL, 100 pmol adapter (10 pmol end-labeled with ^32^P) and 0.5 mM ATP were incubated in 1× adenylation buffer (50 mM MOPS, pH 7.5, 10 mM KCl, 5 mM MgCl_2_) supplemented with 2.5 mM MnCl_2_ and 1 mM DTT for 1 h at 60°C. The reaction was stopped by   heat inactivation for 10 min at 80°C followed by preparative denaturing PAGE and ethanol precipitation. For details on oligomers, see Supplementary Information.

### Sample preparation for *in vivo* lead probing

*In vivo* lead probing was performed as described (27,28,40). Briefly, LB medium was inoculated with an *E. coli* DH5a overnight culture to a starting OD_600_ of 0.06, and cells were grown to an OD_600_ of 0.5 at 37°C. Lead-II-acetate solutions were freshly prepared by mixing 3 volumes of a lead-II-acetate stock solution with 1 volume 4× LB medium and pre-warming to 37°C. After reaching the desired density (OD_600_ 0.5), 20 ml of each main culture were mixed with LB-lead-II-acetate solutions to a final concentration of 75 mM and incubated for 7 min. In Pb^2+^(–) samples, lead-II-acetate was replaced by autoclaved, deionized water (dH_2_O). The reaction was stopped by adding 10 ml ice-cold 500 mM EDTA. Cells were immediately pelleted and RNA was isolated using peqGOLD TriFast^®^ (VWR) according to the manufacturer’s instructions and precipitated from the aqueous phase by adding twice the volume of ice-cold isopropanol. The pellet was resuspended in dH_2_O and incubated with 2 U DNase I (NEB). RNA was recovered by phenol/chloroform extraction and ethanol precipitation (41). Recovered RNA was redissolved in dH_2_O and stored at –80°C.

### Specific adapter ligation

Total RNA was used for 2', 3'-cP mapping via specific adapter ligation with *Ath* RNL K152A D726A and for 5′-OH mapping via 3′ dephosphorylation with T4 PNK R38A and subsequent 5′-OH specific adapter ligation with *Eco* RtcB.

#### 2′, 3′-cP capture

35 ng total RNA were pre-incubated with 20 pmol pre-adenylated 3′ adapter for 5 min at 65°C and immediately put on ice for at least 1 min. Subsequently, the mixture was incubated in 1× reaction buffer (20 mM Tris–HCl, pH 7.5, 5 mM MgCl_2_, 2.5 mM spermidine, 100 μM DTT) and 20% (v/v) PEG8000 with 12 pmol *Ath* RNL K152A D726A for 2 h at 25°C in a volume of 16 μl.  In the ligation reaction, the 2', 3'-cP is converted into a 2'-P group that can interfere with reverse transcription. To remove this obstacle, 10 pmol *S. cerevisiae* tRNA 2'-phosphotransferase Tpt1 and 1 mM NAD were added to the ligation mixture. The volume was adjusted to 20 μl with dH_2_O and reaction buffer and the samples were incubated for 30 min at 30°C. The ligated and 2'-dephosphorylated RNA was recovered using the Monarch^®^ RNA clean up kit (NEB) and used as template for reverse transcription.

#### 5′-OH capture

70 ng total RNA were incubated in 1× PNK buffer (NEB) with 1 mM ATP and 10 pmol T4 PNK R38A for 30 min at 37°C in 15 μl total volume. The 3′ dephosphorylated RNA was recovered with the Monarch^®^ RNA clean up kit (NEB) using the protocol to bind RNA down to 15 nt length. The RNA was eluted in 6 μl dH_2_O and 3 μl thereof were pre-incubated with 20 pmol 5′ adapter for 5 min at 65°C and put on ice for 1 min. Subsequently, the samples were incubated in 50 mM Tris–HCl (pH 7.4), 2 mM MnCl_2_, 100 μM GTP and 20% (v/v) PEG8000 with 50 pmol *Eco* RtcB for 1 h at 37°C in 20 μl total volume. The ligated RNA was recovered again with the Monarch^®^ RNA clean up kit and used for 3′ adapter ligation. To this end, the ligated RNA was mixed with 20 pmol pre-adenylated 3′ adapter, pre-incubated for 5 min at 65°C and put on ice for 1 min. The mixture was incubated in 1× T4 ligase buffer (NEB) and 20% PEG8000 (v/v) with 20 pmol T4 RNL 2 truncated KQ for 2 h at 25°C. The ligated RNA was extracted using the Monarch^®^ RNA cleanup kit and used as template for reverse transcription.

### Reverse transcription

The ligated and recovered RNA from both strategies was reverse transcribed with Superscript IV reverse transcriptase (Thermo). Reactions of 20 μl were set up according to the manufacturer, using RT-Primer for 5′-OH samples. For 2′,3′-cP samples, a biotin-dNTP-Mix (final concentration 500 μM dATP/dGTP/dTTP each, 350 μM dCTP, 150 μM biotin-16-dCTP (Jena Bioscience)) and circularization RT-Primer was used. For both strategies, 20 pmol of the respective primer were used containing trace amounts of the same 5′ labeled primer for product detection. After incubation at 55°C for 10 min, the template RNA in the reaction mixes was degraded by adding NaOH to a final concentration of 250 mM and incubation for 3 min at 95°C. The reaction mix was neutralized with 250 mM HCl, cDNA was extracted and size-selected via preparative denaturing PAGE. A ^32^P-labeled size standard was used to identify cDNA above the size of the used RT-Primer + UMI sequence. The cDNA was eluted from the gel and precipitated with ethanol and 10 μg/ml LPA (Thermo) as carrier. Samples of the 2′,3′-cP strategy were circularized (see below), 5′-OH cDNA was redissolved in 20 μl dH_2_O and directly used for amplification and introduction of flow cell linkers and indices.

### Circularization of cDNA and streptavidin bead cleanup

The cDNA for 2′, 3′-cP library construction, redissolved in 20 μl dH_2_O, was incubated for 2 h at 60°C in 1× adenylation buffer supplemented with 2.5 mM MnCl_2_, 10 mM DTT and 50 μM ATP using 2.5 μM TS2126 RNL (42). The mixture was heat-inactivated at 80°C for 10 min, adjusted to 100 μl with 1× wash/binding buffer (20 mM Tris–HCl, pH 7.5, 1 mM EDTA, 0.5 M NaCl) and directly used for purification with Hydrophilic Streptavidin Magnetic Beads (NEB). Per reaction, 16 μl beads were prepared: the beads were washed three times by resuspending them in 160 μl wash/binding buffer each time and removing the supernatant while placing the tubes on a magnetic rack. The washed beads were resuspended with the circularization reaction mixture and incubated for 20 min at room temperature with careful mixing every 5 min. After incubation, samples were spun down, supernatants were discarded and the beads were resuspended by pipetting. The beads where washed three times with 500 μl wash/binding buffer. Each wash included the following steps: resuspension of the beads by pipetting, brief centrifugation in a desktop centrifuge, again careful resuspension of the bead pellet by pipetting, transfer of the sample to a new tube (this step turned out to be crucial to avoid carry over of non-biotinylated cDNA), using a magnetic rack when removing the supernatants. The beads were finally resuspended in 20 μl dH_2_O each and directly used for amplification and introduction of flow cell linkers and indices.

### Introduction of flow cell linkers and indices

**Figure 1.**
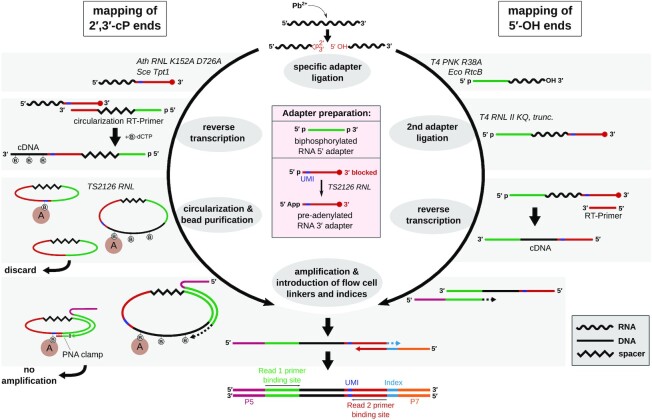
Preparation of Led-Seq Illumina libraries. Left: Mapping of 2′,3′-cP carrying cleavage fragments via specific ligation of a pre-adenylated RNA 3′ adapter using *Ath* RNL AA. The 3′ adapter carries an 8 nt unique molecular identifier (UMI) to account for PCR bias. After ligation, RNA is reverse transcribed using the circularization RT-Primer and biotinylated dCTP (B-dCTP). The resulting cDNA is circularized by TS2126 RNL and extracted with hydrophilic streptavidin magnetic beads (brown circular symbol denoted ‘A’), removing residual circularization RT-Primer in the process. Circularized, bead-bound cDNA is used as a PCR template to introduce flow cell linkers (P5, P7) and 6 nt indices. A PNA PCR clamp is utilized to minimize the amplification of circularized products without cDNA insert. Right: Mapping of 5′-OH carrying cleavage fragments via specific ligation of a biphosphorylated RNA 5′ adapter using *Eco* RtcB. RNA was previously 3′ dephosphorylated with T4 PNK R38A to prevent ligation of RNA fragments with 2′,3′-cP to 5′-OH ends. The 3′ adapter is subsequently ligated to the RNA using T4 RNL II KQ truncated, followed by reverse transcription and amplification via PCR.

Flow cell binding sequences and indices were introduced via PCR. One reaction mix contained 2.5 μl of the cDNA template solution, 1× Phusion HF buffer (Thermo), 200 μM dNTPs, 0,5 μM Illumina PCR and Index Primer and 0.02 U/μl Phusion^®^ high-fidelity polymerase (Thermo) in a volume of 25 μl. To samples containing circularized cDNA templates, 5 μM of a PNA clamp were added to reduce the accumulation of a side product resulting from residual prolonged circularization RT-Primer as template in this reaction. Cycling conditions were as follows: initial denaturation at 98°C for 30 s, followed by 15 (5′-OH strategy) / 18 (2′,3′-cP strategy) cycles of 98°C for 10 s, 80°C for 20 s, 60°C for 20 s and 72°C for 20 s. The additional annealing step at 80°C was introduced to ensure optimal PNA binding in the 2′, 3′-cP libraries (43). The amplified libraries were purified by preparative native PAGE, excised and eluted from the gel, and precipitated with ethanol and 10 μg/ml LPA as carrier. The final libraries were sequenced on an Illumina NovaSeq 6000 (Azenta). The experimental protocol is illustrated in Figure 1.

### Read preprocessing and mapping

Sequencing quality of paired reads was evaluated using MultiQC (44). 3′ adapter sequences were removed from read1 and read2, and pairs were filtered for a correct UMI sequence in read2 with cutadapt v2.10 (45). A minimum read length of 12 nt was set to enable unambiguous mapping. An additional fixed sequence between the UMI and RNA insert was removed from the 5′-end of read2 and if necessary from the 3′-end of read1 by cutadapt. Preprocessed reads were mapped to the *E. coli* genome (NZ_CP026085.1) with segemehl v0.3.4 (46,47). Libraries were deduplicated with umi_tools v1.0.1 (48) and filtered for primary hits of properly mapped read pairs. After an initial sample composition analysis, selected (multi-copy) genes were masked by substitution with ‘N’ and one copy was attached to the end of the genome to facilitate unique mapping of reads. As we wanted to focus our analysis on highly represented transcripts, this procedure included all tRNA and rRNA genes. For details on the generated genome and corresponding transcriptome annotation file, see Supplementary Information. Mapping and deduplication steps were repeated accordingly.

### Intensity of the probing signal

We filtered for reads that mapped uniquely in proper pairs and subsequently considered only hits that mapped to non-overlapping annotated regions (bedtools v2.27.1 (49)) to ensure an unambiguous signal. In the 2′-3′-cP libraries, the last nucleotide of a ligated RNA fragment represents the position immediately upstream of the cleavage site in the RNA backbone. Correspondingly, the first nucleotide of a fragment in 5′-OH libraries represents the position downstream of the cleavage site. The raw probing signal was obtained for each nucleotide of the transcriptome by counting the number of read starts (or start-1, respectively) at that position. Normalization of the raw signal was performed separately for each transcript. According to Low and Weeks (50), we divided the raw read count by the average count of the 90^*th*^ to 98^*th*^ percentile of the signal. This is motivated by considering the largest 2% of the signals as outliers. We denote the normalized signal for position *i* by *S*_*i*_. Its range is limited to the interval [0,7] because very high values of outliers were capped at 7. Where applicable, mean values of replicates were used for all downstream analyses. The workflow for the computation of the normalized probing signal from raw sequencing reads is implemented as a snakemake v3.13.3 pipeline (51), which is available at github.com/xamiiii/Led-Seq.

### Estimating the probability to be unpaired

We use a bayesian approach to estimate the probability *q*_*i*_ that position *i* is unpaired based on the normalized signal *S*_*i*_. To this end, we employ a collection of reference structures comprising 32 RNAs of lengths 74 nt – 682 nt. This set includes non-coding RNAs that belong to Rfam families (52) and that are sufficiently represented by our data, see coverage criteria below. The small-subunit rRNA (16S) and the large-subunit rRNA (23S) were divided into smaller domains as described (53,54). Secondary structures for the resulting 40 sequences were taken from the RNAcentral data base (see Supplementary Table S1 and Supplementary Information for full details).

Denote by *n*_*u*_(*S*) and *n*(*S*), the number of unpaired positions and the total number of positions, respectively, that exhibit a normalized signal in the calibration set that falls within a bin, i.e. an interval of signal values, centered at *S*. The probability that a position with a signal that falls within this bin is unpaired can then be estimated by


(1) $$\begin{equation*} p(S): \mathbb {P}[\rm {unpaired}|S ] \approx \frac{ n_u(S) }{ n(S) } \end{equation*}$$

**Figure 2.**
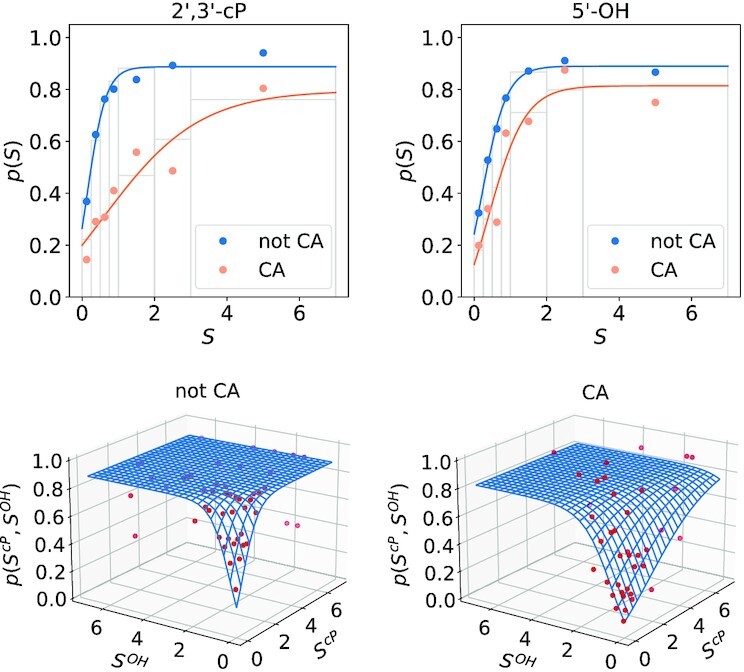
Conversion of normalized probing signals *S* to probabilities of being unpaired. Top: One-dimensional function *p*(*S*) estimated separately for 2′, 3′-cP (left) and 5′-OH libraries (right). Different fits were used for cleavage at CA dinucleotide positions (orange). Grey lines indicate the intervals (‘bins’) within which the signals were pooled. Below: Two-dimensional fit *p*(*S*^*cP*^, *S*^*OH*^) combining the information of both libraries. Again CA dinucleotides (right) were treated separately.

In a more elaborate model, we combine the two signals *S*^*cP*^ and *S*^*OH*^ of the 2′, 3′-cP and 5′-OH libraries. We then consider


(2) $$\begin{equation*} p(S^{cP},S^{OH}): \mathbb {P}[\rm{unpaired}|S ] \approx \frac{ n_u(S^{cP},S^{OH}) }{ n(S^{cP},S^{OH}) } \end{equation*}$$

where *n*_*u*_(*S*^*cP*^, *S*^*OH*^) and *n*(*S*^*cP*^, *S*^*OH*^) are the counts of unpaired and all positions, respectively, in the calibration set that have signal values for the 2′, 3′-cP and 5′-OH libraries within intervals centered at *S*^*cP*^ and *S*^*OH*^, respectively. In order to reduce the effects of inaccuracies in the reference structures and other noise in the data we approximate *p*(*S*) and *p*(*S*^*cP*^, *S*^*OH*^) by fitting sigmoidal functions of the form


(3) $$\begin{eqnarray*} && p(S) = \left( 1+\exp (-aS+b) \right)^{-1} + c \nonumber\\ && \quad p(S^{cP},S^{OH}) = \left(1 + \exp (-a_1 S^{cP} -a_2 S^{OH} + b) \right)^{-1} + c\nonumber\\ \end{eqnarray*}$$

to the binned data, see Figure 2. To estimate parameters *a*, *b*, *c*, and *a*_1_, *a*_2_, *b*, *c*, respectively, we used the function curvefit of the python v3.6.12 library scipy v1.5.2. Since cleavage sites in CA dinucleotides were found to behave differently compared to the other dinucleotides, different parameters were fitted for this special case. As a control we randomized the sequence positions and, as expected, obtained a flat response curve, see Supplementary Figure S1.

The 2′,3′-cP library produces sequence fragments that are too short for reliable mapping close to the 5′ end of each transcript. Consequently, no signal *S*^*cP*^ exists for the first 11 nucleotides of each transcript. Analogously, the 5′-OH library is uninformative close to the 3′ end (last 12 nt). Thus we use $p(S^{OH}_i)$ or $p(S^{cP}_i)$ for positions *i* at the ends of a transcript and $p(S^{cP}_i,S^{OH}_i)$ for its interior.

### Secondary structure prediction with probing data

The conversion of the probability of being unpaired, i.e. *q*_*i*_ = *p*(*S*_*i*_) or $q_i=p(S_i^{cP},S_i^{OH})$, into a pseudo-energy in essence follows the scheme proposed by Zarringhalam *et al.* (55). However, we associate pseudo-energies only with the unpaired nucleotides. It is not difficult to see that it suffices to associate pseudo-energies only with paired or only with unpaired nucleotides as long as one is not interested in the absolute value of the partition function, see (12).

To incorporate the probing data into secondary structure prediction algorithms, we converted $q_{i}$ into pseudo free energy terms that can be interpreted as log-likelihoods for nucleotides being unpaired (56). In addition, we compensate for the fact that the *a priori* probability *p*_0_ that a base is unpaired differs from 1/2. This yields


(4) $$\begin{equation*} \Delta G_{Pb^{2+},i} = -RT \cdot c \cdot \left( \ln \frac{q_{i}}{1-q_{i}} - \ln \frac{p_{0}}{1-p_{0}} \right) \end{equation*}$$

where *R* is the gas constant, *T* is the absolute temperature, and *c* is a constant that allows to tune the relative importance or trust in the probing data. Throughout this contribution, we used *c* = 1.2. The value of *p*_0_ = 0.42 was determined from the calibration set. The position-dependent pseudo-energies are used as soft constraints (12) in the program RNAfold of the ViennaRNA package v2.4.15 (57) as described (11). A detailed structural analysis requires sufficient cleavage signal across the transcript. We require rather stringent conditions: (i) at least 75% of a transcript must be represented by reads, and (ii) there must be at least 2.5 read starts per position on average.

To assess prediction quality, we calculated positive predictive value (PPV), sensitivity (SEN) and the Matthews correlation coefficient (MCC) (definitions see Supplementary Information). Plots were generated using the python package matplotlib v3.3.1. Secondary structures with mapped pseudo-energies were visualized using the forna Web server (58).

## RESULTS

### Lead probing protocol

Metal ion cleavage is an established approach to probe RNA structure (28,59). Recently, Twittenhoff et al. showed that *in vivo* probing with lead(II) ions coupled with next generation sequencing is suitable to investigate RNA structures on a transcriptome-wide level (27). Here, we present a novel lead-based approach where NGS adapters are selectively ligated to the resulting fragments of lead-induced cleavage (see Figure 1). Accordingly, both cleavage ends (2′, 3′-cP and 5′-OH) are converted into sequencing libraries. During the development of this method, we noticed two major side products in the sequencing data of the 2′, 3′-cP libraries, both identified as results from the circularization reaction with TS2126 RNL. These originated from leftover 3′ adapter and circularization RT-Primer in the reverse transcription reaction which subsequently gave rise to the presence of three RT-Primer based DNA species. In addition to circularization RT-Primer+UMI+cDNA, also circularization RT-Primer+UMI and RT-Primer alone were present, despite performing a size selection via preparative denaturing PAGE after reverse transcription. These side products caused a considerable loss in usable sequences. To reduce these side products, we combined two strategies. First, cDNA was internally labeled with biotin-dCTP and extracted using magnetic streptavidin beads after circularization to eliminate unused circularization RT-Primer. Second, as PNA oligonucleotides exhibit a very tight binding to complementary DNA sequences with high thermal duplex stability (43,60), we designed a PNA clamp binding to the constant 3′-region of the UMI-containing oligonucleotide and to the 5′ adapter-derived cDNA sequence. These regions are only adjacent if the circularization RT-Primer elongated by the sequence of the 3′ adapter was circularized without insert. Hence, the PNA clamp blocked the amplification of this side product. Both strategies led to a drastically reduced formation of by-products (see Supplementary Figure S2, Supplementary Figure S3).

**Figure 3.**
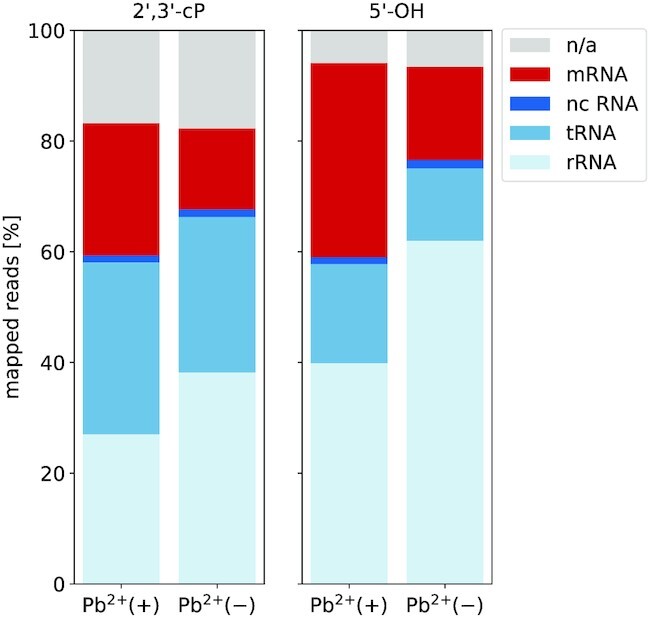
Proportion of reads mapping to indicated RNAs in 2′, 3′-cP (left) and 5′-OH libraries (right) for samples treated with (+) or without (−) Pb^2+^. nc = non-coding, n/a = not annotated

We generated two independent biological replicates of both Pb^2+^(+) libraries. In addition, negative controls in the absence of lead (Pb^2+^(–)) were prepared and sequenced. One of these controls is shown here as a representative example. After read preprocessing, mapping and removal of duplicated reads, we obtained 4.8 – 16.8 million uniquely mapping reads per library (see Supplementary Table S2). Figure 3 summarizes the composition of mapped read ends in terms of the annotated biotypes. As expected, the majority of reads originates from rRNA and tRNAs. The distributions are similar for lead-treated and negative control libraries and also differ only moderately between the 2′, 3′-cP and 5′-OH libraries.

Between 221 and 465 RNAs in the Pb^2+^(+) libraries (and 104 to 171 RNAs in the Pb^2+^(-) libraries) are covered sufficiently by reads to meet our criteria for structural analysis (described in Methods section). Of these, we set up a benchmark set of 32 transcripts that we analyzed in more detail. We found that probing signals are highly reproducible with a Pearson correlation coefficient of 0.83 for the two 2′,3′-cP libraries and 0.80 for 5′-OH libraries (calculated over all sufficiently covered transcripts).

### Signal corresponds to structure

**Figure 4.**
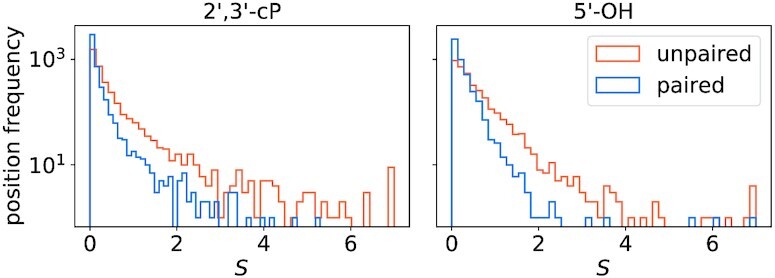
Distribution of normalized probing signal *S* at unpaired (orange) and paired (blue) positions of the calibration set. Signal is higher at unpaired sites in the 2′, 3′-cP (left) and 5′-OH libraries (right).

Based on reference structures, we investigated distributions of the signal for paired and unpaired nucleotides separately (Figure 4). Consistent with our expectations, unpaired positions display significantly higher cleavage levels (two-sided Mann–Whitney U test *p* = 3.0 × 10^−210^ for 2′, 3′-cP and *p* = 2.9 × 10^−200^ for 5′-OH). We also observed that most positions exhibit low signal regardless of the structure. High signal values are therefore informative for unpaired positions. However, not all nucleotides that are unpaired in the reference structures are associated with high cleavage activity. Thus, low signals do not provide a reliable predictor for pairedness.

**Figure 5.**
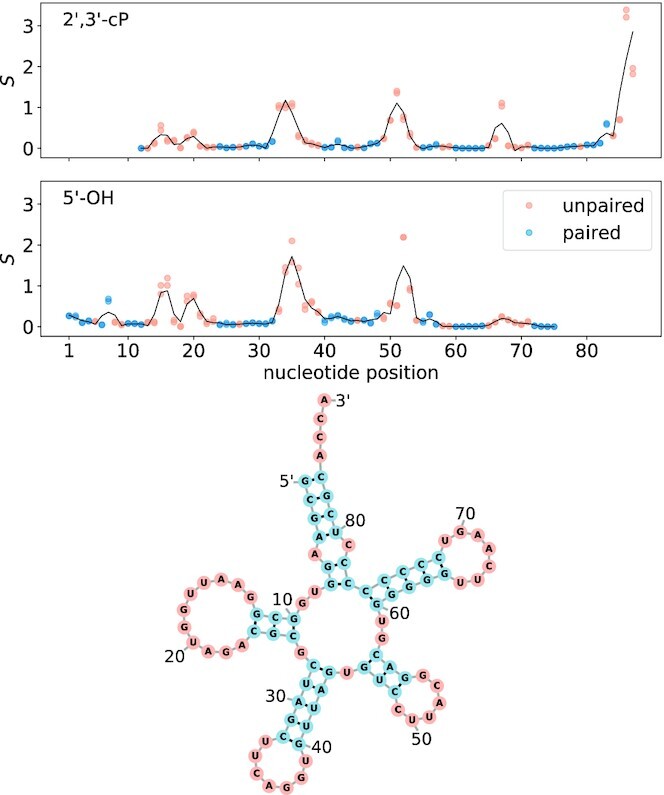
Probing signal profile of tRNA^Leu(CAG)^ reflects the secondary structure. tRNA^Leu(CAG)^ signal profile from (top) 2′, 3′-cP and (bottom) 5′-OH libraries. Depicted are both biological replicates as dots and a savitzky-golay smoothing curve for visualization only. Coloring in orange or blue according to unpaired or paired status within the structural reference.

To visualize the correlation between probing intensity and structure, the profile for tRNA^Leu(CAG)^ is displayed in Figure 5. D-loop, anticodon loop, variable loop, T-loop as well as the unpaired 3′ end consisting of the CCA sequence and the discriminator base are reflected in the peaks of 2′,3′-cP as well as 5′-OH libraries. Since we capture both RNA cleavage fragments in our protocol, the information for the entire transcript is retained even though both library types have one ‘blind’ end of 11 (or 12) nucleotides. To further investigate and quantify the structural information within the probing signal, we converted the scores into probabilities to be unpaired *q*_*i*_, see Figure 2 in the Methods section.

**Figure 6.**
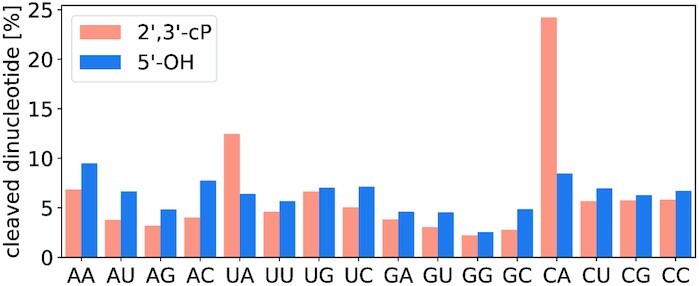
Read fractions (in %) sorted by dinucleotide composition at the cleavage site in 2′,3′-cP and 5′-OH libraries. Adenine – A, uracile – U, guanine – G, cytosine – C.

Since the mapped reads determine the exact cleavage position, we considered the possibility of sequence-specific biases. We indeed found a strong bias towards CA dinucleotides and a milder bias towards UA in 2′,3′-cP libraries (Figure 6). This observation cannot be explained by genomic overrepresentation of these dinucleotides or the influence of only a few outliers (see Supplementary Figure S4). Cleavage between C and A (but not between U and A) is clearly less correlated to an unpaired structural state compared to all other dinucleotides. We account for this effect by estimating the probability of unpairedness *q*_*i*_ separately for CA and all other dinucleotides, see Figure 2 above. The deviations observed for UA in the 2′,3′-cP libraries were small enough to be neglected.

### Comparison of Pb^2+^(+) and Pb^2+^(–) libraries

**Figure 7.**
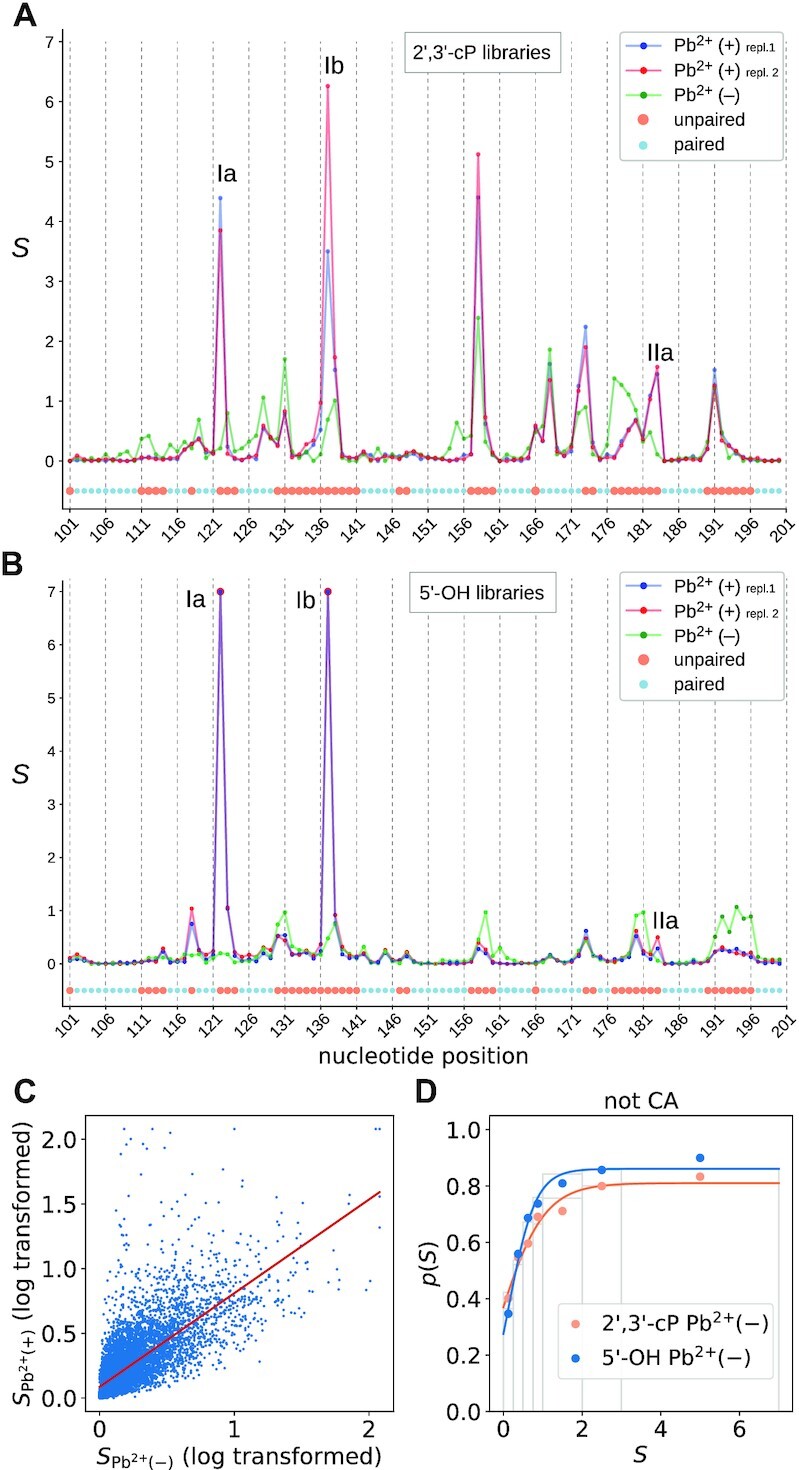
RNase P RNA signal profile (nucleotide positions 101-200) comparing samples treated with (+) or without (–) Pb^2+^ from (**A**) 2′, 3′-cP and (**B**) 5′-OH libraries. The profiles for Pb^2+^-treated samples (Pb^2+^(+), replicate 1 and 2) and for the H_2_O control (Pb^2+^(–)) are superimposed in different colors. Paired and unpaired nucleotides are indicated by small cyan (paired) and large ocher (unpaired) spheres below the profiles according to the structure shown in Supplementary Figure S6A; Roman numerals that mark prominent cleavage sites and nucleotide numbering (x-axis) and as in Supplementary Figure S6A. (**C**) Correlation between probing signals for Pb^2+^(–) and of Pb^2+^(+) 5′-OH libraries in the benchmark set. (**D**) Function *p*(*S*) of Pb^2+^(–) libraries reveals their structural content.

The signal profiles of Pb^2+^-treated and non-treated samples in a subregion of the non-coding RNase P RNA are shown as an example in Figure 7. RNase P RNA harbors several high affinity metal ion binding sites that were identified already in previous studies (26,61,62). Two major (Ia and Ib) and one minor (IIa) high affinity metal ion binding site that are present in the illustrated subregion yielded strong signals in the Pb^2+^-treated but not in the H_2_O control libraries (Figure 7A, B; see Supplementary Figure S6 and accompanying supplementary text for further details). Likewise, Figure 7C illustrates that there are many pronounced peaks in the profiles after lead treatment that are absent in the control samples. These findings emphasize the successful application and utility of lead-induced cleavage in combination with end-specific ligation reactions to determine RNA secondary structure. The high quality of the data is illustrated in detail in Supplementary Figure S6B. We noticed that the Pb^2+^(–) libraries were also highly correlated with both Pb^2+^(+) signal and the unpaired positions of the known reference structures (Figure 7). Overall, the distribution of normalized signals in the H_2_O-treated sample is similar to that of the Pb^2+^-treated samples (Supplementary Figure S5A), although the separation of unpaired and paired positions is less pronounced compared to Figure 4. We find that the patterns are close matches for both the 2′, 3′-cP libraries (Figure 7A) and the 5′-OH libraries (Figure 7B). The normalized signals are also highly correlated across the entire data set (Figure 7C, Supplementary Figure S5B). This similarity between Pb^2+^-treated and H_2_O-treated libraries persists when considering the unpaired positions of our calibration set. As shown in Figure 7D (Supplementary Figure S5C), the Pb^2+^(–) signal can also be converted into a probability *q*_*i*_ that behaves similarly to the one obtained from the Pb^2+^-treated samples. We therefore tested whether an additional ‘bonus energy’ based on Pb^2+^(–) as independent source of information results in an increase of secondary structure inference over the use of Pb^2+^(+) only. Since this was not the case, the negative control libraries were not used in further downstream analysis.

### Experimental signal improves RNA secondary structure prediction

Probing data by definition offer only information whether a given nucleotide is paired or unpaired and therefore provide experimental constraints on RNA secondary structures rather then determining the structure unambiguously. We therefore assessed the usefulness of the Led-Seq data by testing whether they are capable of improving the secondary structures over computational predictions based on the rules of the thermodynamic standard model. To this end, we calculated pseudo-energy contributions for positions with *q*_*i*_ greater than the average probability to be unpaired *p*_0_. These values served as soft constraints for the computation of MFE structures with RNAfold.

**Figure 8.**
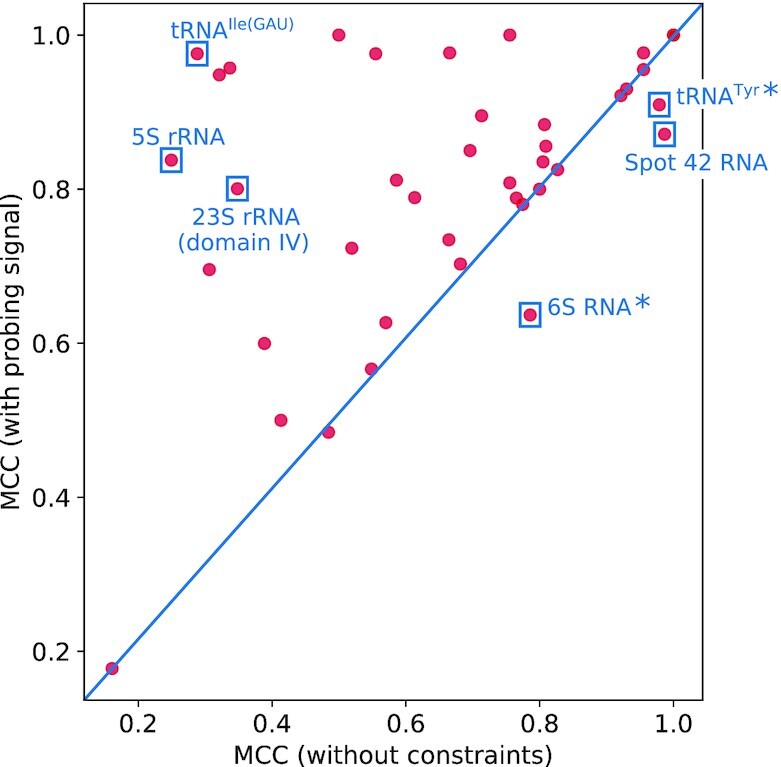
Matthews correlation coefficient (MCC) for structures predicted with or without experimental signal in the form of soft constraints based on both libraries. Known references served as ‘correct’ structures. Dots reflect one transcript of the benchmark set. Positions above the diagonal correspond to improved structure prediction. For 6S RNA and tRNA^Tyr^ (labeled by *), the positions indicate a lower correlation of the mapped structures with the reference structures. This, however, is the result of structural artifacts in the references that do not correspond to experimental findings.

We observed that for the vast majority of the transcripts in our benchmark set there is indeed an improvement. More precisely, the MFE structures computed with the experimentally determined pseudo-energies are close to the reference structures (Figure 8). All reference structures from RNAcentral as well as predictions by construction do not include pseudoknots. Pseudoknots therefore appear as ‘unpaired’ in the reference structures, see e.g. Supplementary Figure S6.

**Figure 9.**
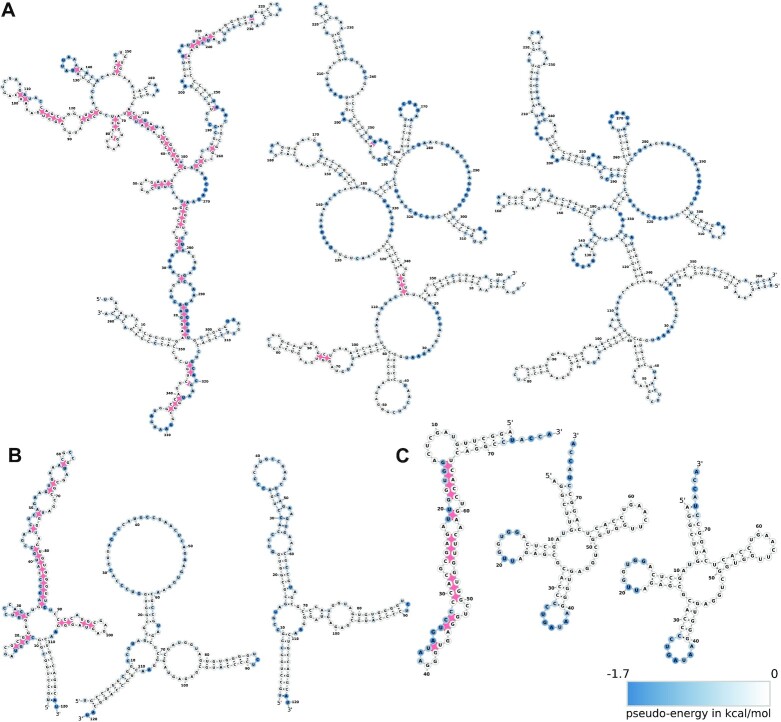
Effect of Led-Seq data on secondary structure prediction of three examples from the calibration data set: (**A**) domain IV of the 23S rRNA, (**B**) 5S rRNA and (**C**) tRNA^Ile(GAU)^. In each case we show the structure predicted by the thermodynamic model (left), the structure obtained by incorporating the probing data as pseudo-energies in RNAfold (middle) and the reference structure taken from the RNAcentral data base. In each case, the incorporation of the probing data pushes the structure closer to the reference. Note that negative pseudo-energies stabilize the *unpaired* state of nucleotide positions and thus led to more open secondary structures. Base pairs that deviate from the reference structure are highlighted in red.

Table 1 summarizes the results quantitatively in terms of the quality metrics PPVs, SENs, and MCCs. Notably, applying the experimental signal of only one type of probing library (2′, 3′-cP or 5′-OH) already yields substantially increased prediction accuracy. This demonstrates the high performance of both libraries individually. As expected, best accuracy is achieved by incorporating both libraries. In particular, improvements close to both ends of a transcript require both libraries since each library type is informative only for either the 5′ or the 3′ terminal region. Figure 9 gives three illustrative examples (5S rRNA, 23S rRNA (domain IV) and tRNA^Ile(GAU)^) showing how incorporation of experimental pseudo-energies guides structure prediction. In particular, positions engaged in false-positive (stacked) base pairs predicted by the thermodynamic model are rearranged towards an open structure upon inclusion of experimental evidence by means of pseudo-energy contributions. Prediction accuracy decreases in only three cases (Figure 8, Supplementary Figure S7). In all three cases, major parts of the mapped structure agree with the reference. Differences are confined to additional base pairs at positions without cleavage signal and unpaired regions in our predictions that are shown as paired in the reference but show large cleavage signals. Considering that the reference structures are derived from computationally obtained consensus structures (63), these three cases can be attributed mostly to issues with the reference structures. In the case of 6S RNA, it is known that upon transcription, this molecule is restructured and forms a hairpin between positions 132 and 152 (64–66) (Supplementary Figure S8), which is clearly visible in our probing analysis. The reference structure, however, does not represent this element. For tRNA^Tyr^, Led-Seq shows an open conformation for the variable loop, while it is base-paired in the reference. As the resulting terminal loop consists of three individual residues, it is very likely that this poses a considerable tension on the short helical part that, due to the central U-A pair, is not highly stable. As a consequence, this region probably shows a certain fraying, resulting in a single-stranded conformation that is recognized by Led-Seq. For more details, see Supplementary Information.

**Table 1.** Assessment of secondary structure prediction accuracy. Minimum free energy (MFE) structures were calculated for all benchmark RNA sequences with and without experimental data as soft constraints. Applying only the signal from 2′, 3′-cP or 5′-OH libraries yields higher prediction quality. Incorporating probing data from both library types achieves highest precision. **PPV** positive predictive value, **SEN** sensitivity, **MCC** Matthews correlation coefficient

	PPV	SEN	MCC
No constraints	0.62	0.70	0.66
Soft constraints - 2′, 3′-cP libraries	0.77	0.78	0.77
Soft constraints - 5′-OH libraries	0.79	0.79	0.79
Soft constraints - both libraries	0.82	0.81	0.81

### mRNA analysis

The secondary structures of mRNAs in the vicinity of the translation start site have repeatedly been reported to be subject to selective pressures. Such effects can be detected by considering patterns of accessibility, i.e. the profile of the position-specific unpairedness *q*_*i*_, see e.g. (23,27,67,68).

**Figure 10.**
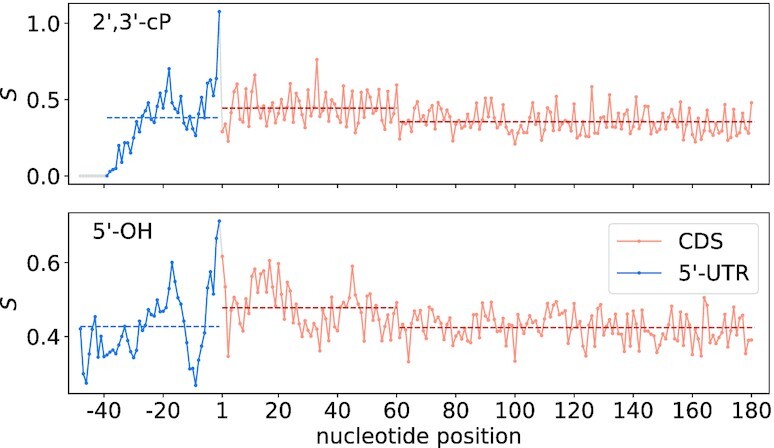
Mean normalized probing signal of coding sequences (CDS) and 5′ untranslated regions (5′-UTR) calculated for mRNAs aligned at start codon from (top) 2′,3′-cP libraries (*n* = 89) and (bottom) 5′-OH libraries (*n* = 146). Included positions range from −48 to 180 with position 1 corresponding to the 5′-position of the AUG start codon. Dashed lines represent mean values for the respective regions.

We collected all mRNAs for which a stretch of 50 nucleotides upstream of the AUG start codon does not intersect another annotated gene and that are sufficiently covered by the probing data (*n* = 89 for 2′,3′-cP libraries and *n* = 146 for 5′-OH libraries). Sequences were aligned at AUG start codons and mean values were calculated for every position in the 5′ untranslated region (UTR, positions −48 to −1) and the beginning of the coding sequence (CDS, positions 1 to 180, 60 codons). As there is no valid signal for the first 11 nucleotides within the 2′,3′-cP libraries, these positions where excluded. The resulting profiles are displayed in Figure 10. The signal shows a pronounced peak at position −1, i.e. just before the start codon, indicating an open conformation at this site. There is a (local) minimum around position −9. This area coincides with the location of the ribosomal binding site (Shine-Dalgarno sequence). Further upstream around position −17, we observe a local maximum. The first 60 nt of CDSs appear to be less structured than 5′-UTRs (2′,3′-cP libraries: mean 5′-UTR = 0.38, mean CDS_1-60_=0.45, two-sided *t*-test *p* = 0.05; 5′-OH libraries: mean 5′-UTR = 0.43, mean CDS_1-60_=0.48, two-sided *t*-test *p* = 0.001). However, after this stretch of 20 codons, the signal drops again to a consistently lower level (2′, 3′-cP libraries: mean CDS_61-180_=0.35, two-sided *t*-test *p* = 2.0 × 10^−9^; 5′-OH libraries: mean CDS_61-180_=0.42, two-sided *t*-test *p* = 6.3 × 10^−11^). We further assessed the mean structural signal of first, second and third positions within codons. Triplets were found to exhibit a significant periodicity in CDSs. No periodicity is detectable in the 5′-UTRs. Both libraries suggest that, on average, last codon positions are less structured than first codon positions (see Supplementary Figure S9).

## DISCUSSION

### Utility and limitations of probing methods for RNA structure elucidation

Here we show that Led-Seq is capable of probing RNA secondary structures *in vivo* with highly reproducible results between biological replicates. A major advantage of the method is that the interrogation of both cleavage products in the 2′,3′-cP and 5′-OH libraries not only increases the reliability of the data and provides an internal quality control, but also ensures that the full length of transcripts can be probed in a high throughput setting.

As with other probing methods, including SHAPE, lead cleavage does not unambiguously distinguish between paired and unpaired positions but provides quantitative evidence that can be converted into a probability that a nucleotide is unpaired. We emphasize that this is not a methodological shortcoming but an inevitable consequence of the fact that RNAs form a free-energy weighted ensemble of structures rather than a single, unambiguous secondary structure (8,69,70). Indeed, recently methods have become available that deconvolve multiple representative structures from a probing signal (70–72). The ‘known’ reference structures are therefore necessarily approximations rather than a perfect gold standard.

The probing signal is confounded further by the fact that the chemical reactions underlying a probing method are influenced by the detailed local conformation of the target, metal ion or protein binding, and other factors that go beyond the pairing status of nucleotides. It is therefore not surprising that the lead probing signal does not distinguish paired from unpaired positions in an all-or-nothing fashion. Instead, one observes distributions of signals *S* that are biased towards larger signals for unpaired positions. The distribution of lead signals in Figure 4 are indeed very similar to corresponding distributions for SHAPE data, see e.g. (73).

A general issue of probing methods is that low signals, here for paired nucleotides, cannot be distinguished from missing data due to other causes, such as limited accessibility to the reagent, see e.g. (74,75). Based on this ambiguity, we cannot safely use low values of *S* and thus *p*(*S*) as support for pairedness. We therefore include pseudo-energies only if *p*(*S*) > *p*_0_, i.e., if the signal provides evidence that a position in unpaired. Lifting this restriction indeed did not improve the prediction quality.

We observed that the incorporation of probing data did not result in improved predictions in a subset of the benchmark structures, and in a few cases the prediction accuracy even decreased, albeit only slightly. This is not unexpected. First, in many cases the thermodynamic model produces rather accurate structures even without additional experimental evidence (76,77). In these cases, additional experimental data confirm rather than modify the structure. Moreover, the reference data from RNAcentral have been obtained with the help of templates that, in turn, are informed either by *in vitro* structures obtained from NMR or X-ray data, or have been constructed as consensus structures over a large set of phylogenetically related molecules (63). They cannot account for fluctuations in the structures of actual RNAs with (temporarily) open segments as implied by probing data of the transcripts with decreased folding accuracy. Taken together, it is reassuring that inclusion of the probing data increases consistency with the reference models. At the same time there is no reason to expect the probing data to reproduce the reference structure perfectly. Figure 8 can thus be interpreted as strong support for the proper functioning and usefulness of Led-Seq.

### Negative controls as information source in Led-Seq

We observed that negative controls without lead treatment (Pb^2+^(–)) produce a signal that is similar to the Pb^2+^(+) libraries. This observation can be explained by the reactive intracellular environment. For example, [Zn(H_2_O)_5_OH]^+^ and [Cu(H_2_O)_5_OH]^+^ with pK_a_ values of 8 to 9 (78) were also shown to be able to hydrolyze *E. coli* RNase P RNA at neutral pH (79). Considering that Zn^2+^ and Cu^2+^ are natural trace elements, it is reasonable to assume that RNA fragments generated by endogenous transition metal ion hydrates entered the 2′,3′-cP and 5′-OH libraries of the H_2_O control samples. Although lead-treated libraries are more informative owing to the identification of additional cleavage sites and a better separation of paired and unpaired signals, the Pb^2+^(–)-libraries also convey structural information. This implies that the Pb^2+^(–) libraries are not genuine background controls, thus questioning the concept to determine differences or ratios such as $S^{\mathrm{Pb^{2+}}}/S^{\mathrm{H_2O}}$ for quantifying the results of *in vivo* lead probing experiments. We emphasize that the lack of a ‘control’, or rather a *background* signal is not an issue that might invalidate the method. In fact, genome-wide data sets often include sufficient information on the background already in the foreground data to render negative controls redundant. In the case of Led-Seq, the relevant information on the background signal is implicitly provided by the distribution of normalized signals for paired positions in the reference structures. We therefore advocate to utilize the control libraries in Led-Seq experiments as an additional source of RNA structure information and to assess the quality and integrity of Led-Seq data by computing the distributions of normalized signals and the functions *p*(*S*) using a few reference structures, preferably well-characterized non-coding RNAs.

### Bias for cleavage between pyrimidine and adenosine in 2′,3′-cP libraries

In general, RNA cleavage induced by Pb^2+^ has been reported to have no overall sequence bias (27). Nevertheless, a considerable bias towards the representation of cleavage sites between CA, and to a lesser extent UA, dinucleotides was evident in our 2′,3′-cP libraries. At present, we can only speculate about the origin of this effect. In several small RNA sequencing libraries, ligation biases were described, and the secondary structures of the ligation partners seem to represent a major cause of such biases (80). In our libraries, however, the CA (UA) bias is presumably not induced by secondary structures, because the remaining three dinucleotides starting with C or U did not show this behavior. In addition, we did not observe any sequence-related effect in ligation efficiency. Theoretically, the observed pattern could also be caused by an endonuclease with a specific recognition sequence that leaves 2′,3′-cP residues after cleavage. *E. coli* expresses such enzymes, most notably MazF (81,82). In a previous study targeting cyclic phosphate-containing RNAs in mice, a similar effect was observed predominantly in mRNA and attributed to a hypothetical enzymatic cleavage event (83). In following studies, the authors reported a similar effect in human cell lines and explained it as the product of ANG cleavage (84). In *Bombyx mori* cells, cleavage with BmRNase κ (85) was identified as a possible cause. If a similar mechanism is at work on *E. coli* RNA, we would expect to observe the overrepresentation of the CA dinucleotide in both, 2′,3′-cP and 5′-OH libraries. This, however, is not the case.

Hence, the molecular basis for this phenomenon is currently unclear. To our knowledge, no bacterial enzyme is known that cleaves RNAs specifically at pyrimidine-A dinucleotides leaving 2′,3′-cP but not 5′-OH groups (82). The effect is also not caused by an increased occurrence of the CA dinucleotide in the *E. coli* transcriptome and our data do not contain one or a few ‘hotspot’ RNAs whose over-representation might explain the CA bias. Interestingly, UA and CA phosphodiester-bonds have been described as more susceptible to hydrolysis in general. However, this effect was later reported to be too small and unsystematic and also highly dependent on the other neighboring nucleotides in the investigated oligomers. Eventually, it was attributed to other structural effects, such as stacking interactions, that enhance or reduce cleavage (86–89). Further investigation is needed to determine the cause for this dinucleotide bias in 2′,3′-cP libraries. From another perspective, our observation of a CA bias in the 2′,3′-cP libraries, but essentially not in the 5′-OH libraries, again illustrates the strength of our dual Led-Seq approach. The observed discrepancy suggests unresolved technical reasons and prevented us from drawing premature conclusions on the biological significance of this finding.

### Analysis of mRNA structures

Led-Seq can also be used to evaluate mRNA structure. Lead treatment of cells resulted in a larger relative abundance of reads mapping to mRNAs in both libraries (Figure 3). The coverage of mRNAs (or other non-coding RNAs) could be improved further by implementing an rRNA depletion step. Since we aimed to demonstrate the general applicability of Led-Seq in this work, we did not include any selection step for certain RNAs, especially since rRNAs and tRNAs are also part of our benchmark set. In first experiments on the applicability of commercially available rRNA depletion kits we could already observe that both RNase H-based (NEBNext^®^, NEB) and bead-based (riboPOOLs, siTOOLs) kits are compatible with our approach and allow a considerable reduction of the rRNA content (data not shown). With the possibility of an individual adaptation of the used probes to other targets, such as tRNAs, we expect a general applicability of these kits for the depletion of a variety of RNAs. Nevertheless, the application of these methods can also bring disadvantages. For example, RNase H-based depletion methods have already been shown to display off-target effects that can negatively impact ribosome profiling data (90). Averaging the normalized probing signals of mRNAs aligned at the start codon (position 1) revealed a local minimum around −9 nt and a local maximum around −17 nt in both libraries. These signals are remarkably consistent with earlier reports based on parallel analysis of RNA structure (PARS) probing data (67). Del Campo et al. postulated that the unstructured region 20 nt upstream of the start codon serves as a non-specific docking site of the 30S ribosomal subunit and described it as a general feature of *E. coli* genes. They interpreted the low signal near nucleotide position -10 as an effect of the Shine-Dalgarno sequence. We also observed a substantially increased signal immediately preceding the start of CDS, implying an open conformation. This observation also conforms to previous findings (67,68,91). Comparison of the average signal intensity of UTRs and CDSs showed a significant increase in the first 60 nt of the CDS.

We also noticed a periodicity of the signal in the mRNA coding regions, while such a signal is absent in the UTR. Interestingly, the 2′, 3′-cP mapping shows a significantly higher signal for every 3rd position of a codon. In contrast, 5′-OH data show no difference between 2nd and 3rd position, but a significantly lower signal for the first position. Taken together, this leads to the conclusion that the third codon position is more susceptible to Pb^2+^-induced cleavage than the first position and thus appears less likely to be ‘structured’. The same effect was already observed in PARS data of *E. coli* (67), as well as in lead-based structure analysis in *Yersinia pseudotuberculosis* (27). Other studies also recognized a periodic pattern in eukaryotic mRNA. However, in DMS-seq data of *A. thaliana* (68), PARS data for *S. cerevisiae* (23), and CIRS-seq data for mice (92), the first nucleotide was least likely to be ‘structured’. Intriguingly, no periodicity was observed in SHAPE-MaP data for *E. coli* (93). The authors provided several explanations for this discrepancy, which we would like to address with an emphasis on our approach: The authors argued that the RNases used in the PARS approach are known to exhibit a certain sequence bias in their cleavage efficiency. In contrast, Pb^2+^-induced cleavage occurs sequence-independent (27). Another potential cause are artifactual signals generated either by methodical or cellular processes. While we cannot rule out a methodical cause entirely, we would not expect to see a periodical pattern restricted to coding regions. Moreover, cotranslational decay by exonucleases as described by Pelechano et al. (94) would result in fragments that are not mapped by our approach. To our knowledge, no known exonuclease leaves 2′,3′-cP and 5′-OH ends. The periodicity effect observable in our data is also not assignable to *in vitro* probing conditions, as we used an *in vivo* probing approach. The only suggested explanation for the mentioned discrepancy between the methods, that applies to Led-Seq, is that it relies on the enzymatic ligation of adapters to cleavage fragments to infer structural information. Therefore it is, in principle, prone to a bias based on the ligation properties of the used enzymes. Despite the enhanced CA dinucleotide cleavage described above, where a ligation bias can be excluded, we could not identify a ligation bias matching the average nucleotide composition of the investigated mRNAs. The periodicity of the genetic code is a known feature based on the fact that in coding sequences no truly random nucleotide/triplet composition is present due to the inherent bias of amino acid-coding triplets (95). Our results suggest that the observed structural periodicity is in fact caused by this intrinsic feature of coding sequences in DNA and therefore in mRNA. It is conceivable that the small size allows the lead ions to enter actively translating ribosomes, where they might have access to the mRNA codons engaged in tRNA binding. The weak wobble interaction at codon position three would then be less protected from cleavage, resulting in the observed periodicity pattern. Further investigation is needed to explain the discrepancy between SHAPE-MaP results and other previous findings concerning CDS structural periodicity.

## CONCLUSION

The Led-Seq approach described here offers the unique advantage that both sites of the metal ion-induced cleavage position in RNA are mapped, increasing the reliability of the observed signals. Furthermore, the interrogation of both cleavage sites allows for the structural analysis of RNA regions close to the 5′ and 3′ ends of transcripts, as the two separate libraries can mutually compensate for information loss at the end of transcripts, which is caused by inaccurate mapping of short cDNAs to the genome. This poses an advantage over other sequencing based methods that lose the 3′-end information because of a missing compensation option. While mutational profiling approaches elegantly circumvent that information loss, RT reactions generally represent an error-prone enzymatic step and inherent RT stops and nucleotide misincorporations may result in a loss of information. Using our double-end approach, we minimize artificially introduced signals by a redundant design of the method. Nonetheless, Led-Seq and SHAPE based approaches complement each other well, as they involve entirely different chemistries. An additional advantage resulting from the use of metal ion-based cleavage is its potential applicability for the *in vivo* investigation of the structurome of psychrophiles and thermophiles, as the exploited probing reaction is theoretically suitable for a wide range of temperatures. Taken together, the double-end structure investigation of Led-Seq represents a very useful approach to characterize RNA structures *in vivo* as well as *in vitro*, expanding our technical arsenal to investigate structure–function relations of RNA.

## DATA AVAILABILITY

The data for this study have been deposited in the European Nucleotide Archive (ENA) at EMBL-EBI under accession number PRJEB58715, see www.ebi.ac.uk/ena/browser/view/PRJEB58715. A computational pipeline is accessible at github.com/xamiiii/Led-Seq and https://doi.org/10.5281/zenodo.7821447.

## Supplementary Material

gkad312_Supplemental_Files

## References

[1] Guerrier-Takada C. , Gardiner K., Marsh T., Pace N., Altman S. The RNA moiety of ribonuclease P is the catalytic subunit of the enzyme. Cell. 1983; 35:849–857. 6197186 10.1016/0092-8674(83)90117-4

[2] Kieft J.S. , Rabe J.L., Chapman E.G. New hypotheses derived from the structure of a flaviviral Xrn1-resistant RNA: conservation, folding, and host adaptation. RNA Biol. 2015; 12:1169–1177. 26399159 10.1080/15476286.2015.1094599 PMC4829329

[3] Serganov A. , Patel D.J. Ribozymes, riboswitches and beyond: regulation of gene expression without proteins. Nat. Rev. Genet. 2007; 8:776–790. 17846637 10.1038/nrg2172 PMC4689321

[4] Vicens Q. , Kieft J.S. Thoughts on how to think (and talk) about RNA structure. Proc. Natl. Acad. Sci. U.S.A. 2022; 119:e2112677119. 35439059 10.1073/pnas.2112677119 PMC9169933

[5] Thirumalai D. , Lee N., Woodson S.A., Klimov D.K. Early events in RNA folding. Annu. Rev. Phys. Chem. 2001; 52:751–762. 11326079 10.1146/annurev.physchem.52.1.751

[6] Turner D.H. , Mathews D.H. NNDB: the nearest neighbor parameter database for predicting stability of nucleic acid secondary structure. Nucleic Acids Res. 2010; 38:D280–D282. 19880381 10.1093/nar/gkp892 PMC2808915

[7] Zuker M. , Stiegler P. Optimal computer folding of larger RNA sequences using thermodynamics and auxiliary information. Nucleic Acids Res. 1981; 9:133–148. 6163133 10.1093/nar/9.1.133 PMC326673

[8] McCaskill J.S. The equilibrium partition function and base pariring probabilities for RNA secondary structures. Biopolymers. 1990; 29:1105–1119. 1695107 10.1002/bip.360290621

[9] Sun L. , Fazal F.M., Li P., Broughton J.P., Lee B., Tang L., Huang W., Kool E.T., Chang H.Y., Zhang Q.C. RNA structure maps across mammalian cellular compartments. Nat. Struct. Mol. Biol. 2019; 26:322–330. 30886404 10.1038/s41594-019-0200-7 PMC6640855

[10] Wang X.-W. , Liu C.-X., Chen L.-L., Zhang Q.C. RNA structure probing uncovers RNA structure-dependent biological functions. Nat. Chem. Biol. 2021; 17:755–766. 34172967 10.1038/s41589-021-00805-7

[11] Lorenz R. , Luntzer D., Hofacker I.L., Stadler P.F., Wolfinger M.T. SHAPE directed RNA folding. Bioinformatics. 2016; 32:145–147. 26353838 10.1093/bioinformatics/btv523 PMC4681990

[12] Lorenz R. , Hofacker I.L., Stadler P.F. RNA folding with hard and soft constraints. Alg. Mol. Biol. 2016; 11:8. 10.1186/s13015-016-0070-z PMC4842303 27110276

[13] Spasic A. , Assmann S.M., Bevilacqua P.C., Mathews D.H. Modeling RNA secondary structure folding ensembles using SHAPE mapping data. Nucleic Acids Res. 2018; 46:314–323. 29177466 10.1093/nar/gkx1057 PMC5758915

[14] Gilmer O. , Quignon E., Jousset A.-C., Paillart J.-C., Marquet R., Vivet-Boudou V. Chemical and enzymatic probing of viral RNAs: From infancy to maturity and beyond. Viruses. 2021; 13:1894. 34696322 10.3390/v13101894 PMC8537439

[15] Mailler E. , Paillart J.-C., Marquet R., Smyth R.P., Vivet-Boudou V. The evolution of RNA structural probing methods: From gels to next-generation sequencing. Wiley Interdiscipl. Rev. RNA. 2019; 10:e1518. 10.1002/wrna.1518 30485688

[16] Strobel E.J. , Yu A.M., Lucks J.B. High-throughput determination of RNA structures. Nat. Rev. Genet. 2018; 19:615–634. 30054568 10.1038/s41576-018-0034-x PMC7388734

[17] Merino E.J. , Wilkinson K.A., Coughlan J.L., Weeks K.M. RNA structure analysis at single nucleotide resolution by selective 2’-hydroxyl acylation and primer extension (SHAPE). J. Am. Chem. Soc. 2005; 127:4223–4231. 15783204 10.1021/ja043822v

[18] Lee B. , Flynn R.A., Kadina A., Guo J.K., Kool E.T., Chang H.Y. Comparison of SHAPE reagents for mapping RNA structures inside living cells. RNA. 2017; 23:169–174. 27879433 10.1261/rna.058784.116 PMC5238792

[19] Kwok C.K. , Tang Y., Assmann S.M., Bevilacqua P.C. The RNA structurome: transcriptome-wide structure probing with next-generation sequencing. Trends Biochem. Sci. 2015; 40:221–232. 25797096 10.1016/j.tibs.2015.02.005

[20] Lu Z. , Chang H.Y. Decoding the RNA structurome. Curr. Op. Struct. Biol. 2016; 36:142–148. 10.1016/j.sbi.2016.01.007 PMC4785074 26923056

[21] Westhof E. , Romby P. The RNA structurome. High-throughput probing. Nat. Methods. 2010; 7:965–967. 21116245 10.1038/nmeth1210-965

[22] Underwood J.G. , Uzilov A.V., Katzman S., Onodera C.S., Mainzer J.E., Mathews D.H., Lowe T.M., Salama S.R., Haussler D. FragSeq. Transcriptome-wide RNA structure probing using high-throughput sequencing. Nat. Methods. 2010; 7:995–1001. 21057495 10.1038/nmeth.1529 PMC3247016

[23] Kertesz M. , Wan Y., Mazor E., Rinn J.L., Nutter R.C., Chang H.Y., Segal E. Genome-wide measurement of RNA secondary structure in yeast. Nature. 2010; 467:103–107. 20811459 10.1038/nature09322 PMC3847670

[24] Forconi M. , Herschlag D. Herschlag D. Metal ion-based RNA cleavage as a structural probe. Biophysical, Chemical, and Functional Probes of RNA Structure, Interactions and Folding. 2009; 468:San Diego, CA Academic Press/Elsevier 91–106. 10.1016/S0076-6879(09)68005-8 20946766

[25] Rubin J.R. , Sundaralingam M. Lead ion binding and RNA chain hydrolysis in phenylalanine tRNA. J. Biomol. Struct. Dyn. 1983; 1:639–646. 6400892 10.1080/07391102.1983.10507471

[26] Ciesiolka J. , Hardt W.D., Schlegl J., Erdmann V.A., Hartmann R.K. Lead-ion-induced cleavage of RNase P RNA. Eur. J. Biochem. 1994; 219:49–56. 8307015 10.1111/j.1432-1033.1994.tb19913.x

[27] Twittenhoff C. , Brandenburg V.B., Righetti F., Nuss A.M., Mosig A., Dersch P., Narberhaus F. Lead-seq: transcriptome-wide structure probing *in vivo* using lead(II) ions. Nucleic Acids Res. 2020; 48:e71. 32463449 10.1093/nar/gkaa404 PMC7337928

[28] Lindell M. , Romby P., Wagner E. G.H. Lead(II) as a probe for investigating RNA structure *in vivo*. RNA. 2002; 8:534–541. 11991646 10.1017/s1355838201020416 PMC1370274

[29] Englert M. , Beier H. Plant tRNA ligases are multifunctional enzymes that have diverged in sequence and substrate specificity from RNA ligases of other phylogenetic origins. Nucleic Acids Res. 2005; 33:388–399. 15653639 10.1093/nar/gki174 PMC546159

[30] Schutz K. , Hesselberth J.R., Fields S. Capture and sequence analysis of RNAs with terminal 2’,3’-cyclic phosphates. RNA. 2010; 16:621–631. 20075163 10.1261/rna.1934910 PMC2822926

[31] Remus B.S. , Shuman S. A kinetic framework for tRNA ligase and enforcement of a 2’-phosphate requirement for ligation highlights the design logic of an RNA repair machine. RNA. 2013; 19:659–669. 23515942 10.1261/rna.038406.113 PMC3677281

[32] Olzog V.J. , Gärtner C., Stadler P.F., Fallmann J., Weinberg C.E. cyPhyRNA-seq: a genome-scale RNA-seq method to detect active self-cleaving ribozymes by capturing RNAs with 2’,3’ cyclic phosphates and 5’ hydroxyl ends. RNA Biol. 2021; 18:818–831. 10.1080/15476286.2021.1999105 PMC8782182 34906034

[33] Chakravarty A.K. , Subbotin R., Chait B.T., Shuman S. RNA ligase RtcB splices 3’-phosphate and 5’-OH ends via covalent RtcB-(histidinyl)-GMP and polynucleotide-(3’)pp(5’)G intermediates. Proc. Natl. Acad. Sci. U.S.A. 2012; 109:6072–6077. 22474365 10.1073/pnas.1201207109 PMC3341019

[34] Peach S.E. , York K., Hesselberth J.R. Global analysis of RNA cleavage by 5’-hydroxyl RNA sequencing. Nucleic Acids Res. 2015; 43:e108. 26001965 10.1093/nar/gkv536 PMC4787814

[35] Solayman M. , Litfin T., Zhou Y., Zhan J. High-throughput mapping of RNA solvent accessibility at the single-nucleotide resolution by RtcB ligation between a fixed 5’-OH-end linker and unique 3’-P-end fragments from hydroxyl radical cleavage. RNA Biol. 2022; 19:1179–1189. 36369947 10.1080/15476286.2022.2145098 PMC9662193

[36] Viollet S. , Fuchs R.T., Munafo D.B., Zhuang F., Robb G.B. T4 RNA ligase 2 truncated active site mutants: improved tools for RNA analysis. BMC Biotech. 2011; 11:72. 10.1186/1472-6750-11-72 PMC3149579 21722378

[37] Blondal T. , Thorisdottir A., Unnsteinsdottir U., Hjorleifsdottir S., Ævarsson A., Ernstsson S., Fridjonsson O.H., Skirnisdottir S., Wheat J.O., Hermannsdottir A.G. et al. Isolation and characterization of a thermostable RNA ligase 1 from a Thermus scotoductus bacteriophage TS2126 with good single-stranded DNA ligation properties. Nucleic Acids Res. 2005; 33:135–142. 15642699 10.1093/nar/gki149 PMC546137

[38] Tanaka N. , Shuman S. RtcB is the RNA ligase component of an Escherichia coli RNA repair operon. J Biol. Chem. 2011; 286:7727–7731. 21224389 10.1074/jbc.C111.219022 PMC3048659

[39] Wang L.K. , Shuman S. Domain structure and mutational analysis of T4 polynucleotide kinase. J. Biol. Chem. 2001; 276:26868–26874. 11335730 10.1074/jbc.M103663200

[40] Ivanova N. , Lindell M., Pavlov M., Holmberg Schiavone L., Wagner E.G.H., Ehrenberg M. Structure probing of tmRNA in distinct stages of trans-translation. RNA. 2007; 13:713–722. 17400816 10.1261/rna.451507 PMC1852820

[41] Sambrook J. , Russell D.W. Purification of nucleic acids by extraction with phenol:chloroform. CSH Protoc. 2006; 2006:1. 10.1101/pdb.prot4455 22485786

[42] Seidl C.I. , Ryan K. Circular single-stranded synthetic DNA delivery vectors for microRNA. PLOS ONE. 2011; 6:e16925. 21359172 10.1371/journal.pone.0016925 PMC3040211

[43] Bender M. , Holben W.E., Sørensen S.J., Jacobsen C.S. Use of a PNA probe to block DNA-mediated PCR product formation in prokaryotic RT-PCR. BioTechniques. 2007; 42:609–614. 17515199 10.2144/000112437

[44] Ewels P. , Magnusson M., Lundin S., Käller M. MultiQC: summarize analysis results for multiple tools and samples in a single report. Bioinformatics. 2016; 32:3047–3048. 27312411 10.1093/bioinformatics/btw354 PMC5039924

[45] Martin M. Cutadapt removes adapter sequences from high-throughput sequencing reads. EMBnet J. 2011; 17:1.

[46] Hoffmann S. , Otto C., Kurtz S., Sharma C., Khaitovich P., Vogel J., Stadler P.F., Hackermüller J. Fast mapping of short sequences with mismatches, insertions and deletions using index structures. PLOS Comp. Biol. 2009; 5:e1000502. 10.1371/journal.pcbi.1000502 PMC2730575 19750212

[47] Hoffmann S. , Otto C., Doose G., Tanzer A., Langenberger D., Christ S., Kunz M., Holdt L.M., Teupser D., Hackermüller J. et al. A multi-split mapping algorithm for circular RNA, splicing, trans-splicing, and fusion detection. Genome Biol. 2014; 15:R34. 24512684 10.1186/gb-2014-15-2-r34 PMC4056463

[48] Smith T.S. , Heger A., Sudbery I. UMI-tools: modelling sequencing errors in unique molecular identifiers to improve quantification accuracy. Genome Res. 2017; 27:491–499. 28100584 10.1101/gr.209601.116 PMC5340976

[49] Quinlan A.R. , Hall I.M. BEDTools: a flexible suite of utilities for comparing genomic features. Bioinformatics. 2010; 26:841–842. 20110278 10.1093/bioinformatics/btq033 PMC2832824

[50] Low J.T. , Weeks K.M. SHAPE-directed RNA secondary structure prediction. Methods. 2010; 52:150–158. 20554050 10.1016/j.ymeth.2010.06.007 PMC2941709

[51] Petereit J. Pipeline automation via snakemake. Methods Mol. Biol. 2022; 2443:181–196. 35037206 10.1007/978-1-0716-2067-0_9

[52] Kalvari I. , Nawrocki E., Argasinska J., Quinones-Olvera N., Finn R., Bateman A., Petrov A.I. Non-coding RNA analysis using the Rfam database. Curr. Protoc. Bioinform. 2018; 62:e51. 10.1002/cpbi.51 PMC6754622 29927072

[53] Jaeger J.A. , Turner D.H., Zuker M. Improved predictions of secondary structures for RNA. Proc. Natl. Acad. Sci. U.S.A. 1989; 86:7706–7710. 2479010 10.1073/pnas.86.20.7706 PMC298139

[54] Mathews D.H. , Sabina J., Zuker M., Turner D.H. Expanded sequence dependence of thermodynamic parameters improves prediction of RNA secondary structure. J. Mol. Biol. 1999; 288:911–940. 10329189 10.1006/jmbi.1999.2700

[55] Zarringhalam K. , Meyer M.M., Dotu I., Chuang J.H., Clote P. Integrating chemical footprinting data into RNA secondary structure prediction. PLOS ONE. 2012; 7:e45160. 23091593 10.1371/journal.pone.0045160 PMC3473038

[56] Cordero P. , Kladwang W., Vanlang C.C., Das R. Quantitative dimethyl sulfate mapping for automated RNA secondary structure inference. Biochemistry. 2012; 51:7037–7039. 22913637 10.1021/bi3008802 PMC3448840

[57] Lorenz R. , Bernhart S.H., Höner zu Siederdissen C., Tafer H., Flamm C., Stadler P.F., Hofacker I.L. ViennaRNA Package 2.0. Alg. Mol. Biol. 2011; 6:26. 10.1186/1748-7188-6-26 PMC3319429 22115189

[58] Kerpedjiev P. , Hammer S., Hofacker I.L. Forna (force-directed RNA): simple and effective online RNA secondary structure diagrams. Bioinformatics. 2015; 31:3377–3379. 26099263 10.1093/bioinformatics/btv372 PMC4595900

[59] Regulski E.E. , Breaker R.R. In-line probing analysis of riboswitches. Methods Mol. Biol. 2008; 419:53–67. 18369975 10.1007/978-1-59745-033-1_4

[60] Giesen U. , Kleider W., Berding C., Geiger A., Ørum H., Nielsen P.E. A formula for thermal stability (*T*_*m*_) prediction of PNA/DNA duplexes. Nucleic Acids Res. 1998; 26:5004–5006. 9776766 10.1093/nar/26.21.5004 PMC147916

[61] Hardt W.D. , Hartmann R.K. Mutational analysis of the joining regions flanking helix P18 in *E. coli* RNase P RNA. J. Mol. Biol. 1996; 259:422–433. 8676378 10.1006/jmbi.1996.0329

[62] Lindell M. , Brännvall M., Wagner E.G., Kirsebom L.A. Lead(II) cleavage analysis of RNase P RNA *in vivo*. RNA. 2005; 11:1348–1354. 16043496 10.1261/rna.2590605 PMC1370818

[63] Sweeney B.A. , Hoksza D., Nawrocki E.P., Ribas C.E., Madeira F., Cannone J.J., Gutell R., Maddala A., Meade C.D., Williams L.D. et al. R2DT is a framework for predicting and visualising RNA secondary structure using templates. Nat. Commun. 2021; 12:3494. 34108470 10.1038/s41467-021-23555-5 PMC8190129

[64] Chen J. , Wassarman K.M., Feng S., Leon K., Feklistov A., Winkelman J.T., Li Z., Walz T., Campbell E.A., Darst S.A. 6S RNA mimics B-form DNA to regulate *Escherichia coli* RNA polymerase. Mol. Cell. 2017; 68:388–397. 28988932 10.1016/j.molcel.2017.09.006 PMC5683422

[65] Beckmann B.M. , Hoch P.G., Marz M., Willkomm D.K., Salas M., Hartmann R.K. A pRNA-induced structural rearrangement triggers 6S-1 RNA release from RNA polymerase in *Bacillus subtilis*. EMBO J. 2012; 31:1727–1738. 22333917 10.1038/emboj.2012.23 PMC3321203

[66] Panchapakesan S. S.S. , Unrau P.J. *E. coli* 6S RNA release from RNA polymerase requires σ^70^ ejection by scrunching and is orchestrated by a conserved RNA hairpin. RNA. 2012; 18:2251–2259. 23118417 10.1261/rna.034785.112 PMC3504675

[67] Del Campo C. , Bartholomäus A., Fedyunin I., Ignatova Z. Secondary structure across the bacterial transcriptome reveals versatile roles in mRNA regulation and function. PLOS Genet. 2015; 11:e1005613. 26495981 10.1371/journal.pgen.1005613 PMC4619774

[68] Ding Y. , Tang Y., Kwok C.K., Zhang Y., Bevilacqua P.C., Assmann S.M. In vivo genome-wide profiling of RNA secondary structure reveals novel regulatory features. Nature. 2014; 505:696–700. 24270811 10.1038/nature12756

[69] Rogers E. , Heitsch C.E. Profiling small RNA reveals multimodal substructural signals in a Boltzmann ensemble. Nucleic Acids Res. 2014; 42:e171. 25392423 10.1093/nar/gku959 PMC4267672

[70] Aviran S. , Incarnato D. Computational approaches for RNA structure ensemble deconvolution from structure probing data. J. Mol. Biol. 2022; 434:167635. 35595163 10.1016/j.jmb.2022.167635

[71] Li T. J.X. , Reidys C.M. On an enhancement of RNA probing data using information theory. Alg. Mol. Biol. 2020; 15:15. 10.1186/s13015-020-00176-z PMC7413225 32782456

[72] Morandi E. , Manfredonia I., Simon L.M., Anselmi F., van Hemert M.J., Oliviero S., Incarnato D. Genome-scale deconvolution of RNA structure ensembles. Nat. Methods. 2021; 18:249–252. 33619392 10.1038/s41592-021-01075-w

[73] Kutchko K.M. , Laederach A. Transcending the prediction paradigm: novel applications of SHAPE to RNA function and evolution: Novel applications of SHAPE. WIREs RNA. 2016; 8:1374. 10.1002/wrna.1374 PMC5179297 27396578

[74] Ingle S. , Azad R.N., Jain S.S., Tullius T.D. Chemical probing of RNA with the hydroxyl radical at single-atom resolution. Nucleic Acids Res. 2014; 42:12758–12767. 25313156 10.1093/nar/gku934 PMC4227780

[75] Solayman M. , Litfin T., Singh J., Paliwal K., Zhou Y., Zhan J. Probing RNA structures and functions by solvent accessibility: an overview from experimental and computational perspectives. Brief. Bioinform. 2022; 23:bbac112. 35348613 10.1093/bib/bbac112 PMC9116373

[76] Hajiaghayi M. , Condon A., Hoos H.H. Analysis of energy-based algorithms for RNA secondary structure prediction. BMC Bioinformatics. 2012; 13:22. 22296803 10.1186/1471-2105-13-22 PMC3347993

[77] Xu X. , Chen S.-J. Physics-based RNA structure prediction. Biophys. Rep. 2015; 1:2–13. 26942214 10.1007/s41048-015-0001-4 PMC4762127

[78] Jackson V.E. , Felmy A.R., Dixon D.A. Prediction of the pKa’s of aqueous metal ion +2 complexes. J. Phys. Chem. A. 2015; 119:2926–2939. 25721568 10.1021/jp5118272

[79] Kazakov S. , Altman S. Site-specific cleavage by metal ion cofactors and inhibitors of M1 RNA, the catalytic subunit of RNase P from *Escherichia coli*. Proc. Natl. Acad. Sci. U.S.A. 1991; 88:9193–9197. 1718000 10.1073/pnas.88.20.9193 PMC52679

[80] Fuchs R.T. , Zhiyi S., Zhuang F., Robb G.B. Bias in ligation-based small RNA sequencing library construction is determined by adaptor and RNA structure. PLOS One. 2015; 10:e0126049. 25942392 10.1371/journal.pone.0126049 PMC4420488

[81] Zhang Y. , Zhang J., Hara H., Kato I., Inouye M. Insights into the mRNA Cleavage Mechanism by MazF, an mRNA Interferase. J.Biol. Chem. 2005; 280:3143–3150. 15537630 10.1074/jbc.M411811200

[82] Bechhofer D.H. , Deutscher M.P. Bacterial ribonucleases and their roles in RNA metabolism. Crit. Rev. Biochem. Mol. Biol. 2019; 54:242–300. 31464530 10.1080/10409238.2019.1651816 PMC6776250

[83] Shigematsu M. , Morichika K., Kawamura T., Honda S., Kirino Y. Genome-wide identification of short 2’,3’-cyclic phosphate-containing RNAs and their regulation in aging. PLOS Genet. 2019; 15:e1008469. 31721758 10.1371/journal.pgen.1008469 PMC6853296

[84] Shigematsu M. , Kirino Y. Oxidative stress enhances the expression of 2’,3’-cyclic phosphate-containing RNAs. RNA Biol. 2020; 17:1060–1069. 32397797 10.1080/15476286.2020.1766861 PMC7549721

[85] Shigematsu M. , Kawamura T., Morichika K., Izumi N., Kiuchi T., Honda S., Pliatsika V., Matsubara R., Rigoutsos I., Katsuma S. et al. RNase κ promotes robust piRNA production by generating 2’,3’-cyclic phosphate-containing precursors. Nat. Commun. 2021; 12:4498. 34301931 10.1038/s41467-021-24681-w PMC8302750

[86] Kierzek R. Hydrolysis of oligoribonucleotides: influence of sequence and length. Nucleic Acids Res. 1992; 20:5073–5077. 1408823 10.1093/nar/20.19.5073 PMC334286

[87] Ciesiołka J. , Michałowski D., Wrzesinski J., Krajewski J., Krzyzosiak W.J. Patterns of cleavages induced by lead ions in defined RNA secondary structure motifs. J. Mol. Biol. 1998; 275:211–220. 9466904 10.1006/jmbi.1997.1462

[88] Mikkola S. , Kaukinen U., Lönnberg H. The effect of secondary structure on cleavage of the phosphodiester bonds of RNA. Cell Biochem. Biophys. 2001; 34:95–119. 11394443 10.1385/CBB:34:1:95

[89] Kaukinen U. , Venäläinen T., Lönnberg H., Peräkylä M. The base sequence dependent flexibility of linear single-stranded oligoribonucleotides correlates with the reactivity of the phosphodiester bond. Org. Biomol. Chem. 2003; 1:2439–2447. 12956059 10.1039/b302751a

[90] Zinshteyn B. , Wangen J.R., Hua B., Green R. Nuclease-mediated depletion biases in ribosome footprint profiling libraries. RNA. 2020; 26:1481–1488. 32503920 10.1261/rna.075523.120 PMC7491325

[91] Burkhardt D.H. , Rouskin S., Zhang Y., Li G.-W., Weissman J.S., Gross C.A. Operon mRNAs are organized into ORF-centric structures that predict translation efficiency. eLife. 2017; 6:e22037. 28139975 10.7554/eLife.22037 PMC5318159

[92] Incarnato D. , Neri F., Anselmi F., Oliviero S. Genome-wide profiling of mouse RNA secondary structures reveals key features of the mammalian transcriptome. Genome Biol. 2014; 15:491. 25323333 10.1186/s13059-014-0491-2 PMC4220049

[93] Mustoe A.M. , Busan S., Rice G.M., Hajdin C.E., Peterson B.K., Ruda V.M., Kubica N., Nutiu R., Baryza J.L., Weeks K.M. Pervasive regulatory functions of mRNA structure revealed by high-resolution SHAPE probing. Cell. 2018; 173:181–195. 29551268 10.1016/j.cell.2018.02.034 PMC5866243

[94] Pelechano V. , Wei W., Steinmetz L. Widespread co-translational RNA decay reveals ribosome dynamics. Cell. 2015; 161:1400–1412. 26046441 10.1016/j.cell.2015.05.008 PMC4461875

[95] Shabalina S.A. , Ogurtsov A.Y., Spiridonov N.A. A periodic pattern of mRNA secondary structure created by the genetic code. Nucleic Acids Res. 2006; 34:2428–2437. 16682450 10.1093/nar/gkl287 PMC1458515

