# Supplementary material for: Led-Seq: ligation-enhanced double-end sequence-based structure analysis of RNA
Source: Nucleic Acids Res. 2023 Apr 28;51(11):e63. doi: 10.1093/nar/gkad312 (PMC10287922; doi:10.1093/nar/gkad312)
Supplement: gkad312_Supplemental_Files [file gkad312_supplemental_files.zip › Supplementary_information.pdf]

Led-Seq – ligation-enhanced double-end sequence-based structure analysis of RNA

Tim Kolberg[†], Sarah von Löhneysen[†], Iuliia Ozerova, Karolin Wellner, Roland K. Hartmann, Peter F. Stadler and Mario Mörl[*]

1 Quality assessment of predicted structures and visualization

To evaluate the quality of predicted structures, we applied three metrics: First, the positive predictive value (PPV), which is the proportion of correctly predicted base pairs in all predicted base pairs. Secondly, the sensitivity (SEN) that is defined as the number of correctly predicted base pairs divided by all base pairs in the reference structure. And the Matthews correlation coefficient (MCC), that combines both:

$$\mathrm{MCC} = \frac{\mathrm{TP}\cdot\mathrm{TN} - \mathrm{FP}\cdot\mathrm{FN}}{\sqrt{(\mathrm{TP}+\mathrm{FP})\,(\mathrm{TP}+\mathrm{FN})\,(\mathrm{TN}+\mathrm{FP})\,(\mathrm{TN}+\mathrm{FN})}}$$

Defined as true positives (TP) are only base pairs (i, j) that occur in the reference and the predicted structure. False positives (FP) are base pairs that are only present in the predicted structure but not the reference. Base pairs in the reference but not the predicted structure are classified as false negative (FN). True negatives (TN) are all base pairs that are not formed in the reference and the predicted structure.

[*]To whom correspondence should be addressed. Tel: +49 (0)341 9736911; Fax: +49 (0) 9736919; Email: mario.moerl@uni-leipzig.de
[†]These authors contributed equally to this work.

Supplementary Table 1: **Benchmark data set.** Given are sequence lengths, the source of reference structures from `RNAcentral` and the download date.

**Sequence**	**Length [nt]**	`RNAcentral` **ID**	**Download date**
5S ribosomal RNA	120	URS0000049E57_562	11/12/2020
23S ribosomal RNA (I)	513	URS0000D1EBB7_562	11/12/2020
23S ribosomal RNA (II)	682	URS0000D1EBB7_562	11/12/2020
23S ribosomal RNA (III)	372	URS0000D1EBB7_562	11/12/2020
23S ribosomal RNA (IV)	362	URS0000D1EBB7_562	11/12/2020
23S ribosomal RNA (V)	605	URS0000D1EBB7_562	11/12/2020
23S ribosomal RNA (VI)	262	URS0000D1EBB7_562	11/12/2020
16S ribosomal RNA (I)	559	URS00003884C2_562	11/12/2020
16S ribosomal RNA (II)	354	URS00003884C2_562	11/12/2020
16S ribosomal RNA (III)	484	URS00003884C2_562	11/12/2020
16S ribosomal RNA (IV)	144	URS00003884C2_562	11/12/2020
$tRNA^{Ile(GAU)}$	77	URS00000FF435_511145	14/12/2020
$tRNA^{Ala(GGC)}$	76	URS00001C39A4_511145	14/12/2020
$tRNA^{Thr(UGU)}$	76	URS00003A295A_511145	14/12/2020
$tRNA^{Leu(CAG)}$	87	URS00005E3C63_316385	15/12/2020
$tRNA^{Met(CAU)}$	77	URS0000093D11_511145	15/12/2020
$tRNA^{Phe}$	76	URS0000099A18_562	15/12/2020
$tRNA^{Gly(GCC)}$	76	URS00001ACE3E_511145	15/12/2020
$tRNA^{Arg(ACG)}$	77	URS0000349C05_511145	15/12/2020
$tRNA^{Glu}$	76	URS000037E2FF_511145	15/12/2020
$tRNA^{Lys}$	76	URS000042DDAB_511145	15/12/2020
$tRNA^{Val(UAC)}$	76	URS000009F4BF_511145	15/12/2020
$tRNA^{Asn}$	76	URS000008CEF9_511145	16/12/2020
$tRNA^{Gln(CUG)}$	75	URS00004FF598_511145	16/12/2020
$tRNA^{Asp}$	77	URS000009EE82_511145	16/12/2020
$tRNA^{Pro}$	77	URS00002E063F_316385	15/12/2020
$tRNA^{Ser}$	93	URS000016F1C4_511145	15/12/2020
$tRNA^{Cys}$	74	URS000003E908_511145	16/12/2020
$tRNA^{Tyr}$	85	URS00004C383B_511145	16/12/2020
$tRNA^{His}$	76	URS0000597E49_511145	16/12/2020
$tRNA^{Trp}$	76	URS00003A6DC6_511145	16/12/2020
CsrB RsmB RNA	360	URS00004CC624_562	04/01/2021
Spot 42 RNA	119	URS0000165C8D_562	04/01/2021
sroB RNA	83	URS000036FEA7_562	04/01/2021
GcvB RNA	206	URS0000257340_562	04/01/2021
cspA thermoregulator	461	URS0000CD8CB8_83333	04/01/2021
tmRNA	363	URS000037602E_511145	14/12/2020
SRP RNA	97	URS00005833D6_511145	04/01/2021
RNase P RNA	377	URS00004BB8BB_511145	12/01/2023
6S RNA	184	URS00003146CE_511145	04/01/2021

Supplementary Table 2: Statistical analysis of sequencing libraries

**Library**	**Total reads**	**Reads with UMI and insert ≥ 12 nt**	**Deduplicated uniquely mapped reads**
2′,3′-cP Pb^{2+}(+) Biological replicate 1	5.6x10^7	3.4x10^7	8.8x10^6
2′,3′-cP Pb^{2+}(+) Biological replicate 2	3.9x10^7	2.7x10^7	8.4x10^6
2′,3′-cP Pb^{2+}(–)	3.0x10^7	2.3x10^7	4.8x10^6
5′-OH Pb^{2+}(+) Biological replicate 1	3.9x10^7	3.1x10^7	1.3x10^7
5′-OH Pb^{2+}(+) Biological replicate 2	4.9x10^7	4.1x10^7	1.7x10^7
5′-OH Pb^{2+}(–)	3.6x10^7	2.8x10^7	1.1x10^7

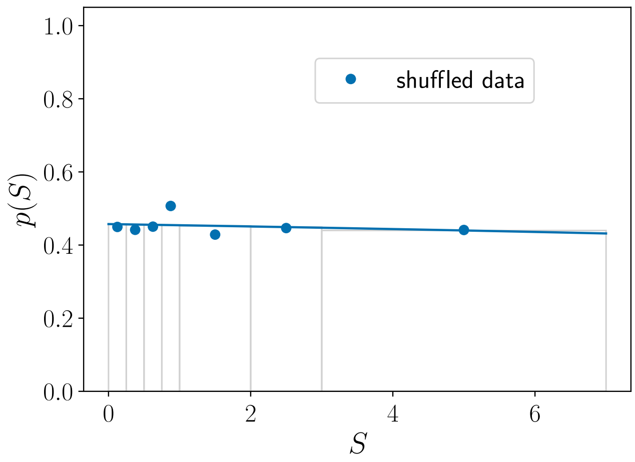

Supplementary Figure 1: One-dimensions functions $p(S)$ after randomizing the signal position, i.e. the signal is detached from its original location in the transcripts. In this negative control there is no correlation between signal intensity S and the probability to be unpaired $p(S)$.

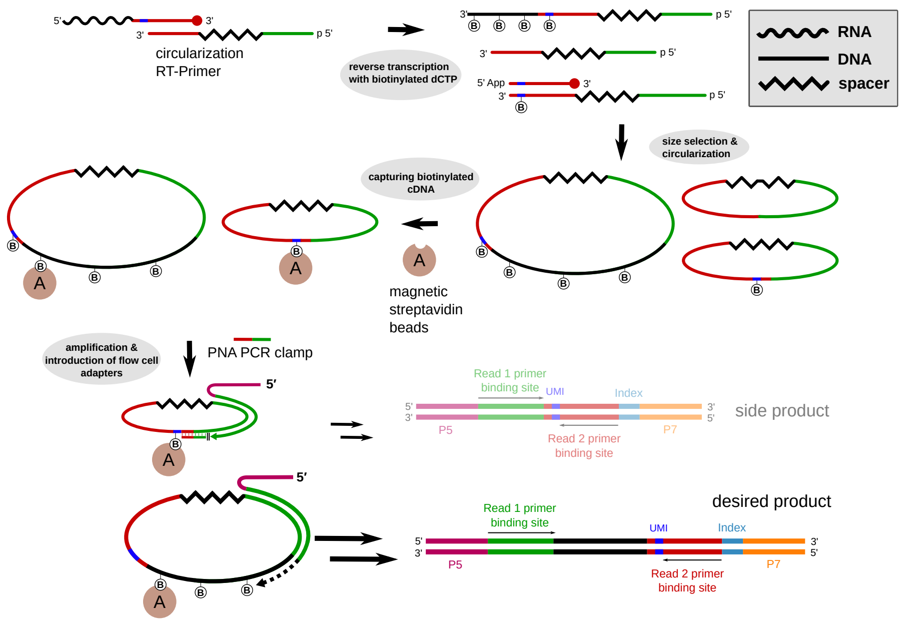

Supplementary Figure 2: While preparing 2′,3′-cP libraries we realized that the circularization of cDNA leads to the accumulation of side products caused by the later amplification of circularized RT-Primer and circularized RT-Primer + UMI sequence. We therefore implemented two strategies to reduce the formation of these side products. To eliminate remaining circularization RT-Primer we used biotinylated dCTP in the reverse transcription reaction to allow the purification of cDNA via magnetic streptavidin beads. This step leaves the desired cDNA products as well as RT-Primer prolonged only by the UMI sequence in the reaction mix. To prevent the amplification of the latter in the final library preparation step, we added a PNA oligomer to the PCR mix. This PNA clamp is complementary to the ligation site of the circularization reaction if there is no RNA derived cDNA insert, preventing side product formation but not the amplification of the desired product.

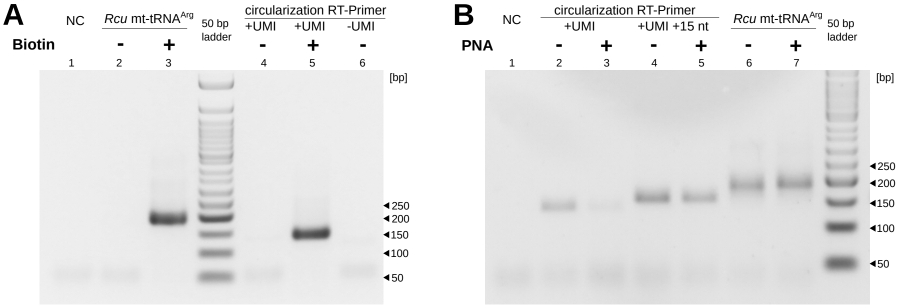

Supplementary Figure 3: Reduction of identified side products in $2',3'$-cP libraries. *In vitro* transcribed mt-tRNA^{Arg} from *Romanomermis culicivorax* (42 nt) was used as control RNA, circularization RT-Primer with and without UMI was used to demonstrate the successful reduction of side products. Library preparation was carried out as described in the method section for $2',3'$-cP libraries if not stated otherwise. Shown pictures are final libraries on 2% agarose gels and negative controls without template (NC). **(A)** Removal of circularized RT-Primer as template. Reverse transcription of adapter-ligated tRNA with circularization RT-Primer was performed with (3) and without (2) biotinylated dCTP. Additionally, remaining circularization RT-Primer (6) was also selectively extracted from the preparative PAA gel, as well as circularization RT-Primer with added UMI sequence (with (5) and without (4) incorporated biotin.) After gel extraction and circularization, magnetic streptavidin beads were used to pull down biotinylated cDNA. The beads were directly used as template for amplification and introduction of flow cell linkers (12 cycles), with the result that only biotinylated cDNA was amplified $(3, 5)$. **(B)** Reduction of amplification of circularized RT-Primer + UMI. Circularization RT-Primer + UMI $(2, 3)$, circularization RT-Primer + UMI + 15 random nucleotides $(4, 5)$ and cDNA of mt-tRNA^{Arg} $(6, 7)$ were circularized and used as template for amplification. If the PNA clamp was added to the reaction mix, it led to reduced amplification of circularized RT-Primer + UMI (3), while the other cDNAs $(5, 7)$ were still amplified.

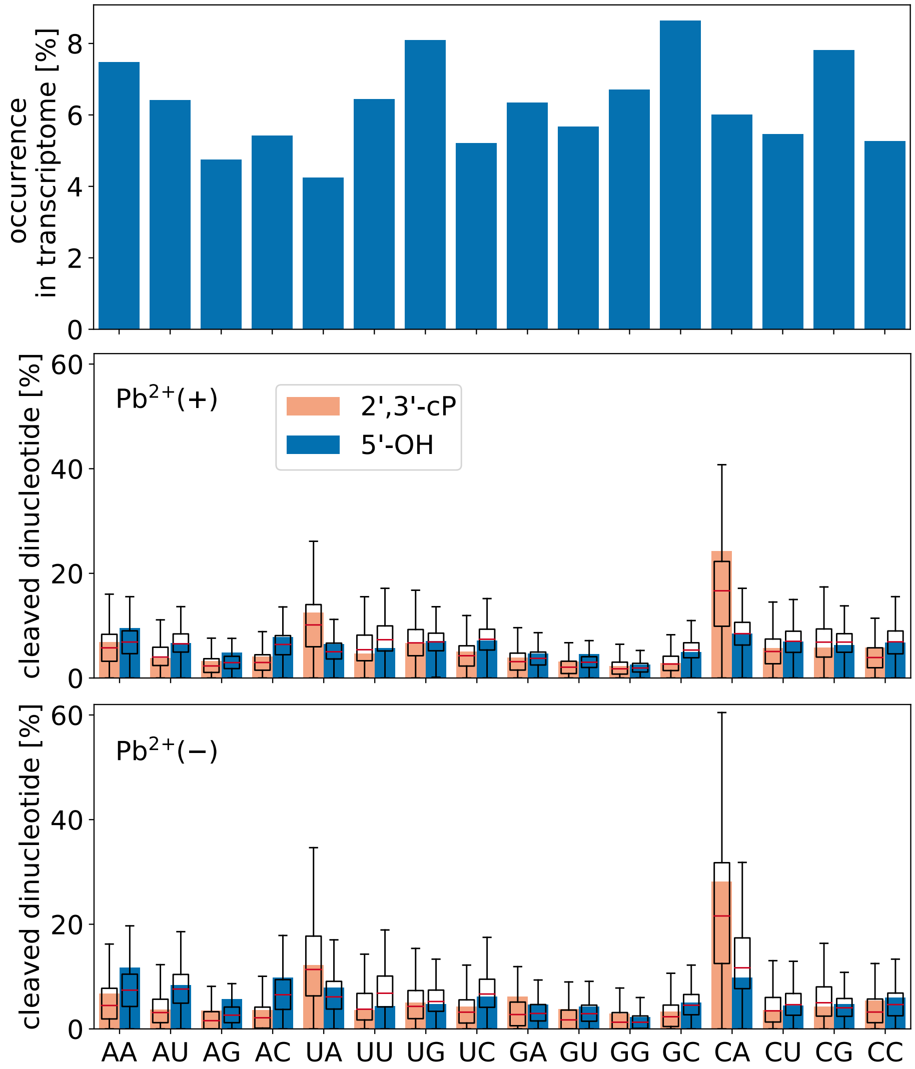

Supplementary Figure 4: **(Top)** Distribution of dinucleotides formed by adenine (A), uracile (U), guanine (G) and cytosine (C) within the *Escherichia coli* transcriptome. **(Middle)/(Bottom)** Transcriptome-wide read fractions (in %) sorted by dinucleotide identity at the cleavage site. Boxplots represent the distribution of the metric when calculated for each transcript individually. Medians are displayed in red, whiskers show data within $Q1-1.5\times IQR$ and $Q3+1.5\times IQR$ with IQR = interquartile range, $Q1(Q3) = 25^{th}(75^{th})$ percentile. Results are shown for libraries treated with lead ($Pb^{2+}(+)$) and with H_2O ($Pb^{2+}(-)$).

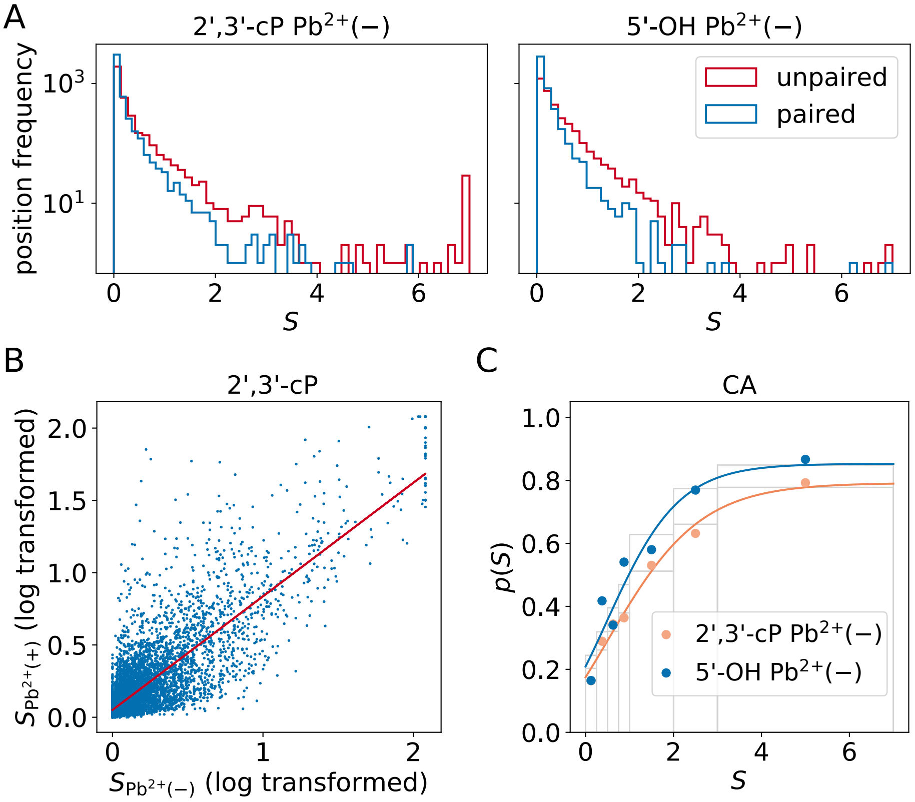

Supplementary Figure 5: Statistics on Pb^{2+}(–) samples. **(A)** Distribution of normalized probing signal at unpaired (red) and paired (blue) positions of the calibration set. Signal is higher at unpaired sites in the 2′,3′-cP (left) and 5′-OH library (right). **(B)** Correlation between probing signal in the benchmark set of Pb^{2+}(–) and of Pb^{2+}(+) 2′,3′-cP libraries. **(C)** Function $p(S)$ of Pb^{2+}(–) libraries at CA cleavage sites.

2 Led-Seq signal of RNase P RNA confirms successful lead structure probing

RNase P RNA is informative in terms of high affinity metal ion binding sites that result in prominent Pb^{2+} ion-induced cleavages [2, 3, 4]. The most prominent cleavages were previously identified 3′ of positions 122 and 137 (site Ia and Ib in Supplementary Figure 6A). Indeed, the normalized probing signals at these positions were essentially the strongest signals for RNase P RNA in Pb^{2+}(+) samples, but were close to background levels in Pb^{2+}(−) control samples, see Supplementary Figure 6B. This confirms particularly efficient Pb^{2+}-induced cleavage at high affinity metal ion binding sites, in line with previous findings, and further demonstrates that our *in vivo* Pb^{2+} probing method worked as intended. Elevated signals in Pb^{2+}(+) samples but substantially lower signals in Pb^{2+}(−) control samples were also observed at lead cleavage sites IIa (nt 183) and IVb (nt 284). Cleavage at site IVb occurs when RNase P is complexed with tRNAs [2, 4]. Supplementary Figure 6B also provides an example for signal overrepresentation of cleavages between C and A in 2′,3′-cP libraries: for hydrolysis between positions C158 and A159, a strong signal was observed in the 2′,3′-cP libraries, particularly in the Pb^{2+}(+) samples, while the signal was quite weak in the 5′-OH libraries. Hydrolysis between C158 and A159 was also observed in an *in vivo* lead probing study using reverse transcription stops on the RNA as readout [4]. There are two other hydrolysis sites between C and A nucleotides in *E. coli* RNase P RNA. One is between C247 and A248 (site IIc, Supplementary Figure 6A). Here, the normalized cleavage signal was about two-fold higher in the 2′,3′-cP versus 5′-OH libraries and signal heights were identical between Pb^{2+}(+) and Pb^{2+}(−) samples, see Supplementary Figure 6B. At site IIb, cleavage between C226 and A227 was detected in 2′,3′-cP libraries, with signal height in Pb^{2+}(+) samples exceeding that in the control sample; in the 5′-OH libraries, the main signal represented cleavage between A227 and C228 (Supplementary Figure 6B). Thus, cleavage between C226/A227 was underrepresented in the 5′-OH libraries or overrepresented in 2′,3′-cP libraries; the latter interpretation would be consistent with the observation that Pb^{2+}-induced hydrolysis between C226/A227 appeared less prominent than hydrolysis between A227/C228 in the study by Lindell et al.[4].

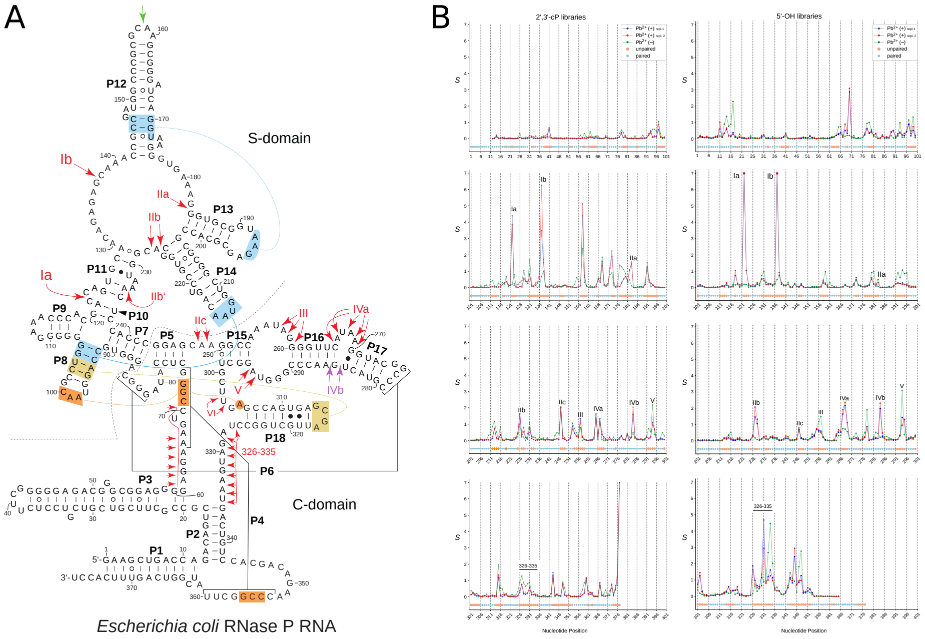

Supplementary Figure 6: Correlation of Led-Seq data for *E. coli* RNase P RNA (gene *rnpB*) with Pb^{2+}-induced hydrolysis sites previously mapped by different analysis methods. **(A)** Secondary structure of *E. coli* RNase P RNA (for more details, see [1]) with previously mapped lead cleavage sites [2, 3, 4] indicated by red arrows (for hydrolysis sites between nt 62 to 70, the mapping accuracy was $\pm$ 1 nt [3]). The gray dashed line separates the catalytic (C-) and specificity (S-) domains. Long-range tertiary interactions are depicted by colored boxes connected by dotted lines, with intradomain contacts highlighted in light blue and interdomain contacts in orange and ocher; adapted from [1]. The green arrow (C158/A159) marks a site that was observed in an *in vivo* probing analysis [4] but not *in vitro*. Roman numerals mark prominent cleavage sites, the strongest are Ia and Ib. Hydrolysis site IVb is shown in magenta, as it is specific for RNase P RNA in complex with tRNAs [2]. **(B)** Normalized probing signals (S) in 2′,3′-cP and 5′-OH libraries that map to *E. coli* RNase P RNA. The profiles for Pb^{2+}-treated samples ($Pb^{2+}(+)$, replicate 1 and 2) and for the H_2O control ($Pb^{2+}(-)$) are superimposed in different colors. Paired and unpaired nucleotides are indicated by small cyan (paired) and large ocher (unpaired) spheres below the profiles according to the structure shown in panel A; nucleotide numbering (x-axis) as in panel A.

3 Structure Prediction without Improvement Relative to the Reference

For three reference structures in Figure 8 the inclusion of Leq-Seq data led to a decrease in prediction accuracy relative to the reference structure. For **tRNA$^{\mathbf{Tyr}}$**, Supplementary Figure 7C, the difference between the MFE and the reference structure is a single A-U base pair at the end of the D-loop that is shown as paired in the MFE predictions and unpaired in the reference. Led-Seq data provide no evidence either way. We observe a pronounced signal for an unpaired state of the variable loop, which is shown as paired in the reference, indicating the region is at least very flexible and partially unpaired.

The **6S RNA** structure, Supplementary Figure 7A, and the **Spot 42 RNA**, Supplementary Figure 7B, show much larger discrepancies. As argued below, these stem from issues with the reference structures rather than a failure of the Led-Seq protocol.

3.1 Secondary Structure of 6S RNA

The cryo-EM structure of *E. coli* RNA polymerase (σ^{70}-RNAP) in complex with *E. coli* 6S RNA [5] supports the structure shown in Supplementary Figure 8A. The structure of free 6S RNA in solution is assumed to be an ensemble of near-isoenergetic structures that are similar to the structure in the complex with RNAP (Supplementary Figure 8A) and the thermodynamic model shown in Supplementary Figure 7A (structure on the left). *E. coli* 6S RNA levels are relatively low during exponential growth phase and peak toward stationary phase [6]. The σ^{70}-RNAP enzyme can utilize 6S RNA as a template for abortive transcripts (termed pRNAs for product RNAs) [7]. In stationary phase, nutrients including NTPs are scarce and RNAP synthesizes very short pRNAs ($\lesssim$8 nt) that rapidly dissociate from 6S RNA. During exponential phase, NTP levels are high and σ^{70}-RNAP synthesizes longer pRNAs of $\geq$13 nt on 6S RNA as template. Such pRNAs remain stably bound to 6S RNA and cause a structural rearrangement of 6S RNA that leads to its dissociation from RNAP [8, 9]. As part of the refolding, an extended hairpin (nt 132-152) forms in the central region of the RNA (Supplementary Figure 8B). This hairpin structure is predicted correctly when our probing data are incorporated into the thermodynamic model as pseudo-energies (Supplementary Figure 7A, middle). This finding suggests that the bulk of 6S RNA was in the refolded state, which nicely fits the fact that the Led-Seq experiments were performed with exponentially grown cells and thus in the presence of high NTP levels. The pRNA:6S RNA hybrid helix, could, of course, not be predicted, as the thermodynamic model for 6S RNA does not include a second trans-acting RNA. However, when the binding site for a pRNA 13-mer is constrained in RNAfold ('must not pair'), the extended hairpin is predicted with high probability. Our findings illustrate that our Led-Seq approach, beyond improving RNA structure predictions in the majority of cases, is also capable of unveiling biologically relevant structural

changes of RNA molecules.

3.2 Secondary Structure of Spot 42 RNA

A secondary structure supported by nuclease and lead probing was proposed by [10], Supplementary Figure 7B (lower right). This model, however, shows rather implausible isolated base pairs in the first stem-loop. The lead cleavage signals throughout the first stem-loop suggest that this region is likely flexible and is not well described by a single secondary structure. Moreover, other aspects may have contributed to the structure model that incorporates the probing data (Supplementary Figure 7B, structures in the middle). Spot 42 RNA acts as an antisense RNA on at least nine mRNA targets and these interactions primarily involve the 5′-proximal region of Spot 42 RNA including the apical loop (nt 32-34 in Supplementary Figure 7B, upper right) [11]. The apical loop region might be an initiation point in the formation of antisense-target interactions, thus frequently adopting a more open conformation. On the other hand, the apical loop region is expected to be inaccessible in most antisense-target RNA complexes [11]. As Led-Seq provides average data, other approaches are required to disentangle the functionally relevant conformational states adopted by Spot 42 RNA *in vivo*. As such, our analysis pipeline has unveiled discrepancies between the current thermodynamic structure model and the conformational equilibria of Spot 42 RNA in growing *E. coli* cells.

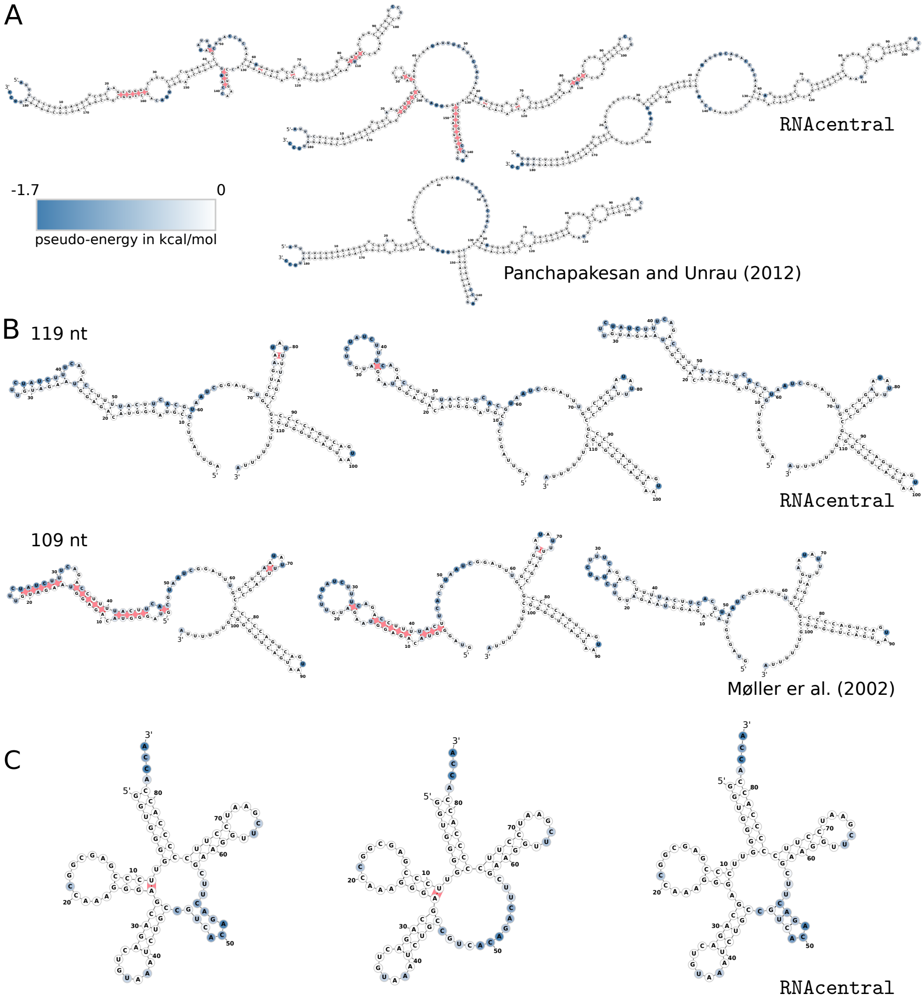

Supplementary Figure 7: Effect of Led-Seq data on secondary structure prediction of **(A)** 6S RNA, **(B)** Spot 42 RNA, and **(C)** tRNA^{Tyr}. For each case we show the structure predicted by the thermodynamic model (left), the structure obtained by incorporating the probing data as pseudo-energies in `RNAfold` (middle) and the reference structure taken from `RNAcentral` (right). The pseudo-energy for unpaired bases derived from the Led-Seq data is indicated by a blue shade. Base pairs that deviate from the reference structure are highlighted in red. For 6S RNA, **(A)**, the experimentally supported structure of [9] (lower middle) is in excellent agreement with the Led-Seq based prediction. For Spot 42 RNA, **(B)**, `RNAcentral` gives a reference structure of length 119 nt, elongated at the 5′ end. Experimental data, however, only support a shorter molecule of length 109 nt, shown below, using the structure model of [10] as reference.

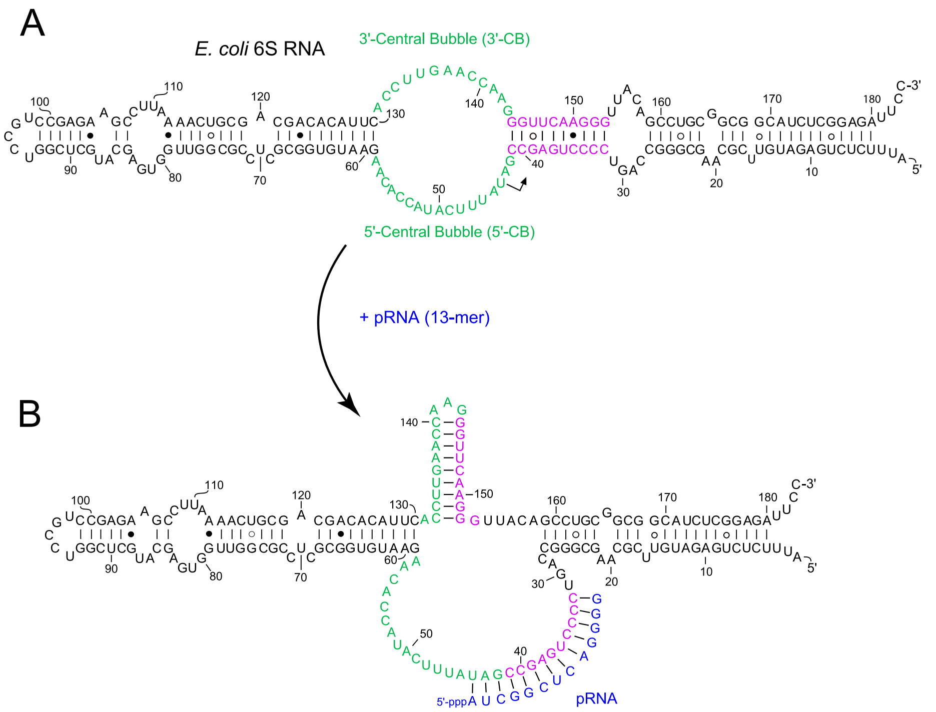

Supplementary Figure 8: Illustration (adapted from [12]) of pRNA-induced refolding of *E. coli* 6S RNA [9]. **(A)** Ground state structure of *E. coli* 6S RNA including structural details derived from its cryo-EM structure in complex with *E. coli* RNA polymerase (σ^{70}-RNAP) [5]. The central bubble is shown in green, the helix that is disrupted during refolding is depicted in pink, and the pRNA transcription initiation site (position 44) is indicated by the rectangular arrow. **(B)** Refolding of 6S RNA upon synthesis of a pRNA 13-mer (in blue) by σ^{70}-RNAP utilizing 6S RNA as template.

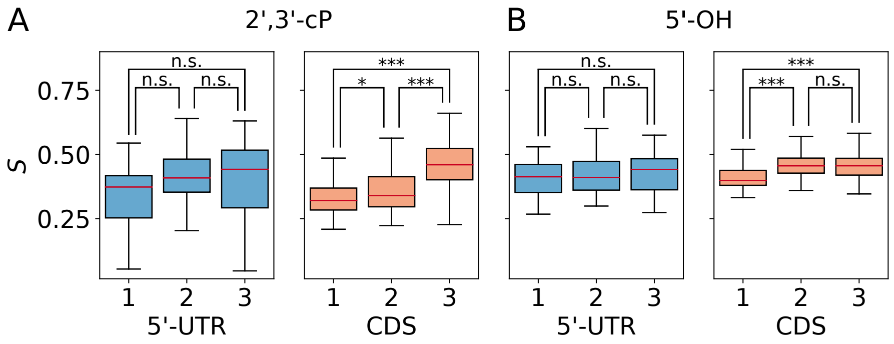

Supplementary Figure 9: Normalized probing signal for all first, second and third positions of each codon in **(A)** 2′,3′-cP libraries and **(B)** 5′-OH libraries calculated for mRNAs aligned at start codon ($n = 89$ and $n = 146$ respectively). Included positions range from -48 to -1 (5′-UTR, 5′-OH libraries) or -39 to -1 (5′-UTR, 2′,3′-cP libraries) and 1 to 180 (CDS) regarding AUG. Distributions are given as boxplots with medians in red, whiskers show data within $\mathrm{Q1} - 1.5 \times \mathrm{IQR}$ and $\mathrm{Q3} + 1.5 \times \mathrm{IQR}$ with IQR = interquartile range, Q1(Q3) = $25^{\mathrm{th}}(75^{\mathrm{th}})$ percentile. Statistical significance calculated by two-sided t-test (n.s. $p > 0.05$; * $p \leq 0.05$; *** $p \leq 0.001$).

References

[1] Schencking, I., Rossmanith, W., and Hartmann, R. K. (2020) Diversity and evolution of RNase P In P Pontarotti, (ed.), Evolutionary biology – a transdisciplinary approach, p. 255–299 Springer Cham.

[2] Ciesiolka, J., Hardt, W. D., Schlegl, J., Erdmann, V. A., and Hartmann, R. K. (1994) Lead-ion-induced cleavage of RNase P RNA *Eur J Biochem.* **219(1-2)**, 49–56.

[3] Hardt, W. D. and Hartmann, R. K. (1996) Mutational analysis of the joining regions flanking helix P18 in *E. coli* RNase P RNA *J Mol Biol.* **259(3)**, 422–433.

[4] Lindell, M., Brännvall, M., Wagner, E. G., and Kirsebom, L. A. (2005) Lead(II) cleavage analysis of RNase P RNA *in vivo RNA* **11(9)**, 1348–1354.

[5] Chen, J., Wassarman, K. M., Feng, S., Leon, K., Feklistov, A., Winkelman, J. T., Li, Z., Walz, T., Campbell, E. A., and Darst, S. A. (2017) 6S RNA mimics B-form DNA to regulate *Escherichia coli* RNA polymerase *Mol Cell.* **68(2)**, 388–397.

[6] Wassarman, K. M. and Storz, G. (2000) 6S RNA regulates *E. coli* RNA polymerase activity *Cell* **101(6)**, 613–623.

[7] Wassarman, K. M. and Saecker, R. M. (2006) Synthesis-mediated release of a small RNA inhibitor of RNA polymerase *Science* **314(5805)**, 1601–1603.

[8] Beckmann, B. M., Hoch, P. G., Marz, M., Willkomm, D. K., Salas, M., and Hartmann, R. K. (2012) A pRNA-induced structural rearrangement triggers 6S-1 RNA release from RNA polymerase in *Bacillus subtilis EMBO J.* **31**, 1727–1738.

[9] Panchapakesan, S. S. S. and Unrau, P. J. (2012) *E. coli* 6S RNA release from RNA polymerase requires σ^{70} ejection by scrunching and is orchestrated by a conserved RNA hairpin *RNA* **18**, 2251–2259.

[10] Møller, T., Franch, T., Udesen, C., Gerdes, K., and Valentin-Hansen, P. (2002) Spot 42 RNA mediates discoordinate expression of the *E. coli* galactose operon *Genes & Dev.* **16**, 1696–1706.

[11] Bækkedal, C. and Haugen, P. (2015) The spot 42 RNA: A regulatory small RNA with roles in the central metabolism *RNA Biol.* **12(10)**, 1071–1077.

[12] Steuten, B., Hoch, P. G., Damm, K., Schneider, S., Köhler, K., Wagner, R., and Hartmann, R. K. (2014) Regulation of transcription by 6S RNAs: insights from the *Escherichia coli* and *Bacillus subtilis* model systems *RNA Biol.* **11(5)**, 508–521.
